# Multimodal Optical Imaging of Ex Vivo Fallopian Tubes to Distinguish Early and Occult Tubo-Ovarian Cancers

**DOI:** 10.3390/cancers16213618

**Published:** 2024-10-26

**Authors:** Jeanie Malone, Adrian S. Tanskanen, Chloe Hill, Allan Zuckermann Cynamon, Lien Hoang, Calum MacAulay, Jessica N. McAlpine, Pierre M. Lane

**Affiliations:** 1 Department of Integrative Oncology, British Columbia Cancer Research Institute, 675 W 10th Avenue, Vancouver, BC V5Z 1L3, Canadaplane@bccrc.ca (P.M.L.); 2 School of Biomedical Engineering, University of British Columbia, 251-2222 Health Sciences Mall, Vancouver, BC V6T 1Z3, Canada; 3 School of Engineering Science, Simon Fraser University, 8888 University Drive, Burnaby, BC V5A 1S6, Canada; 4 Department of Pathology and Laboratory Medicine, University of British Columbia and Vancouver General Hospital, 910 West 10 Avenue, Vancouver, BC V5Z 1M9, Canada; 5 Department Obstetrics and Gynaecology, Division Gynecologic Oncology, University of British Columbia and BC Cancer, 2775 Laurel St, 6th Floor, Vancouver, BC V5Z 1M9, Canada

**Keywords:** optical coherence tomography, autofluorescence imaging, ovarian cancer, endoscopic imaging, falloposcopy, cancer morphology, optical biopsy

## Abstract

**Background:** There are currently no effective screening measures to detect early or occult tubo-ovarian cancers, resulting in late-stage detection and high mortality. This work explores whether an optical imaging catheter can detect early-stage tubo-ovarian cancers or precursor lesions where they originate in the fallopian tubes. **Methods:** This device collects co-registered optical coherence tomography (OCT) and autofluorescence imaging (AFI). OCT provides three-dimensional assessment of underlying tissue structures; autofluorescence imaging provides functional contrast of endogenous fluorophores. Ex vivo fallopian tubes (n = 28; n = 7 cancer patients) are imaged; we present methods for the calculation of and analyze eleven imaging biomarkers related to fluorescence, optical attenuation, and OCT texture for their potential to detect tubo-ovarian cancers and other lesions of interest. **Results:** We visualize folded plicae, vessel-like structures, tissue layering, hemosiderin deposits, and regions of fibrotic change. High-grade serous ovarian carcinoma appears as reduced autofluorescence paired with homogenous OCT and reduced mean optical attenuation. Specimens containing cancerous lesions demonstrate a significant increase in median autofluorescence intensity and decrease in Shannon entropy compared to specimens with no lesion. Non-cancerous specimens demonstrate an increase in optical attenuation in the fimbriae when compared to the isthmus or the ampulla. **Conclusions**: We conclude that this approach shows promise and merits further investigation of its diagnostic potential.

## Simple Summary

Tubo-ovarian cancers are associated with high mortality as they are often not detected until a late stage. Early diagnosis is associated with better patient outcomes, but there are currently no effective screening measures. We explore whether an optical imaging catheter can detect early or occult lesions in the fallopian tubes. This device collects three-dimensional structural images of tissue through optical coherence tomography (OCT) simultaneously with functional imaging through autofluorescence imaging (AFI). We image ex vivo fallopian tubes from n = 28 patients (n = 7 cancer patients) and explore eleven imaging biomarkers for their ability to distinguish early or occult disease. We find that high-grade serous ovarian carcinomas can be visually distinguished through this approach, and that there are several quantitative changes within the area of lesion and throughout the specimen that can be measured through these imaging biomarkers. We conclude that this approach shows promise and merits further investigation of its diagnostic potential.

## 1. Introduction

Patients with tubo-ovarian cancers who are diagnosed with early-stage disease have dramatically improved outcomes when compared to patients with advanced-stage carcinoma [1]. However, there are currently no effective screening tools for this disease site that enable earlier detection [2,3,4,5]. This work explores whether an optical imaging catheter can detect early-stage tubo-ovarian cancers or precursor lesions, focusing on high-grade serous ovarian carcinoma (HGSOC) and serous tubal intraepithelial carcinoma (STIC), which originate in the fallopian tubes [6,7,8]. The lack of adequate screening tools has led to opportunistic salpingectomy programs for primary prevention of tubo-ovarian cancers in average-risk patients, encouraging patients to consider removal of the fallopian tubes at the time of pelvic surgery for other indications [9,10]. High-risk patients, such as those with hereditary indications (e.g., *BRCA1* or *BRCA2* mutations), are recommended risk-reducing bilateral salpingo-oophorectomy (RRBSO) after completion of childbearing or at the age of 35–40, causing early menopause with possible significant long-term health consequences [11]. Novel early detection strategies that enable fallopian tube screening may support a delay in risk-reducing definitive surgical procedures.

Proximally to distally, the fallopian tubes are divided into the intramural (connected to the uterine ostia), the isthmus, the ampulla, and the infundibulum. The infundibulum is funnel-shaped and opens into the peritoneal cavity, fringed with finger-like projections called fimbriae that extend toward the ovary. Microscopically, the fallopian tube consists of an innermost endosalpinx (mucosa) surrounded by the muscularis (myosalpinx), which in turn is surrounded by serosa. The endosalpinx is folded longitudinally to form plicae, which are most complex and pronounced in the distal fallopian tube. The epithelium of the fallopian tubes is a single layer of columnar epithelium which contains secretory epithelial cells in the isthmus, transitioning to ciliated epithelial cells in the ampulla and infundibulum. The myosalpinx consists of layers of muscle arranged in alternating directions. The serosa is composed of the mesothelium of visceral peritoneum and contains vasculature (supplied by the uterine and ovarian arteries), innervation, and lymphatics [12,13,14,15,16]. The fallopian tubes are highly vascularized as previously visualized by confocal microtomography [16,17] and photoacoustic microscopy [18,19].

There is increasing evidence that HGSOC originates in the epithelium of the fallopian tube fimbriae [8,20,21]. Precursor lesions may be microscopic in size and heterogeneously distributed. The diagnostic gold standard for tubo-ovarian cancers is a specialized histologic protocol (‘Sectioning and Extensively Examining the Fimbriated End’, SEE-FIM) that allows for detailed review of the entire fallopian tube and especially the fimbriae, as classical methods have been shown to under-sample and underdiagnose small regions of invasion or STIC [22,23]. Conventional medical imaging techniques lack the resolution to examine the fallopian tubes for microscopic lesions: transvaginal ultrasound, even when combined with blood tests measuring cancer antigen 125 (CA-125), cannot detect disease early enough to reduce deaths due to tubo-ovarian cancers [3,5]. Endoscopic examination of the fallopian tubes (falloposcopy) was popular in the 1990s for fertility assessment prior to our understanding of the role of the fallopian tubes in tubo-ovarian cancers [24]. Regardless, early falloposcopy approaches relied on fiber bundles, which similarly lacked the resolution and contrast to identify small regions of lesion. Recently, advanced falloposcopy device approaches have begun to be explored for potential tubo-ovarian cancer detection [25,26,27,28,29].

Optical imaging techniques use light to provide high-resolution visualizations of tissue at a limited depth of penetration. This can be delivered to luminal organs such as the fallopian tubes endoscopically through fiber optics, allowing for detailed examination of inner lumen surfaces. We have previously developed a multimodal optical imaging catheter capable of co-registered optical coherence tomography (OCT) and autofluorescence imaging (AFI) which can image regions up to 16 cm in length [30,31]. We hypothesize that OCT-AFI will provide utility in early tubo-ovarian cancer detection and present an imaging study demonstrating this technique in ex vivo fallopian tubes.

OCT produces volumetric images of tissue by scanning a low-coherence beam of light across a sample and interfering the collected backscattered light with a path-length-matched reference beam [32]. This allows for the visualization of subsurface morphology, which has been precisely correlated to histology [33], and has been explored in cancer applications for preoperative diagnosis and intraoperative detection of malignancies [34]. While OCT has primarily found clinical adoption in ophthalmology, there is a growing body of work focused on endoscopic applications [35,36]. Endoscopic techniques have a reduced lateral resolution compared to microscopic OCT systems capable of sub-micron resolution [37]; conventional endoscopic OCT has lateral resolutions on the order of 10–40 μm.

OCT has been identified as a potentially useful adjunct in many gynecologic applications including fertility assessment, investigation of chronic inflammatory conditions, and cancer screening [38]. This has included laparoscopic or ex vivo imaging of the exterior of fallopian tubes [39,40,41,42] as well as falloposcopy [25,26,27]. Recently, the first in vivo OCT falloposcopy imaging of healthy volunteers has been demonstrated, collecting long two-dimensional images via manual retraction of an imaging catheter [27]. In OCT, the fallopian tubes appear largely homogenous, and the single cell layer of epithelium is indistinct. Plicae are visualized as folded and often overlapping structures [26,27]. Edema and fibrosis appear as regions of low and high intensity OCT, respectively [39]. Depending on the imaging depth of the OCT system, the mucosa, musculature, and even peritoneal tissue may be distinguishable [25,26]. External imaging of fallopian serosa has demonstrated vessel-like structures as regions of low intensity [40] which appear similar in morphology to micro-focus computed tomography images of fallopian vasculature [12].

As the fallopian tubes and ovaries are complex in structure, distinguishing the most disease-relevant characteristics is challenging. Quantitative image processing extensions have been explored to improve tissue-specific contrast. One such approach is the depth-resolved optical attenuation coefficient, which may capture changes in tissue composition [43,44]. The attenuation coefficient has been demonstrated to be lower in regions of HGSOC compared to surrounding non-cancerous tissue [41]. In addition to the intensity of OCT, subtle textural changes may encode information about the extracellular matrix (collagen remodelling) and other microstructural properties [45]. Haralick texture features [46], which have been used in various medical imaging applications, have demonstrated promise in ex vivo ovarian cancer applications [47,48]. However, OCT texture is also subject to inherent speckle, which itself encodes sub-wavelength scattering information [49]. Quantitative analysis of speckle distribution has demonstrated diagnostic potential in a cervical cancer mouse xenograft model [50].

OCT lends itself well to combination with additional imaging modalities such as fluorescence imaging to improve diagnostic potential [25,27,28,30,31]. Fluorescence imaging may examine the endogenous fluorophores (autofluorescence imaging, AFI) in tissue or may use contrast dyes. Blue-light AFI is used clinically in oral, bronchoscopic, and colposcopic applications [51,52,53,54,55]. It has been demonstrated ex vivo that clinically occult lesions can be detected via AFI in ovarian and fallopian tissues [56,57,58,59,60]. Blue-light AFI captures responses from a variety of biological sources, but predominantly is driven by characteristics of the extracellular matrix. In cancers, collagen remodelling and epithelial thickening result in lower fluorescence response [61].

Achieving multimodal imaging on a scale that allows for the cannulation of the fallopian tubes is challenging. Our approach using double-clad fiber (DCF) to allow for the co-registered collection of both OCT and AFI comes at a cost to each modality. The dopants in DCF contribute additional background fluorescence compared to pure-silica-core single-mode fibers, which reduces the signal to background ratio of the AFI [62]. In DCF-based OCT, near-infrared light may be coupled into higher-order modes which can introduce multipath artifacts. These artifacts appear as ghost images smeared in the A-line direction that may superimpose the fundamental image and cause a reduction in the usable depth of the OCT [31,63,64,65]. However, the benefits of co-registered structural (OCT) and functional (AFI) examination of tissue may outweigh these costs.

## 2. Materials and Methods

### 2.1. Study Design

This work explores whether OCT-AFI will provide utility in early tubo-ovarian cancer detection and analyzes ex vivo imaging of the fallopian tubes to identify prospective image biomarkers that distinguish lesions. The biomarkers described in this work were selected based on features that had demonstrated potential in the previous literature exploring fallopian and ovarian imaging. This includes functional features such as autofluorescence intensity, optical attenuation derived from OCT, and OCT texture features. Due to the small sample size, this is a hypothesis-generating study intended to demonstrate feasibility, identify trends, and provide future directions for diagnostic criteria. This work will explore the following questions:

- Is there a statistical difference in these measurements between disease states, within an individual image, or between images of patients with a lesion/without a lesion? Can these biomarkers be used to visualize areas of lesion?
- In non-lesion cases, are there statistical differences in these measurements in different regions of the fallopian tube?
- How repeatable are these measurements? Are there differences between the left and right fallopian tubes in patients when paired imaging is acquired?
- Are there statistical correlations with age/other patient demographics that might be confounders?

#### 2.1.1. Inclusion Criteria

Any patient undergoing salpingectomy at the Vancouver General Hospital was eligible for this study. Patients must have consented to the British Columbia Gynecologic Tissue Bank (UBC BCCA REB# H05-60119) as well as to this study (UBC BCCA REB #H17-01716). Patients were recruited with a preference for those with known or suspected ovarian cancer (HGSOC) based on clinical history and CA125 levels, although all samples were analyzed regardless of histotype.

#### 2.1.2. Exclusion Criteria

Patients that had undergone chemotherapy prior to salpingectomy were excluded from this study. Samples that were imaged more than two hours after arrival at the pathology department were excluded, as extended ischemic time results in changes in tissue properties and a reduction in autofluorescence response. Imaging deemed of insufficient quality (excessive bubbles or non-uniform rotational distortion; tissue contact for <50% of the image length; poor reference selection, overlaying the desired image with multipath artifacts) was not included in this study.

### 2.2. Imaging System

Images were acquired with a previously described endoscopic OCT–autofluorescence imaging (OCT-AFI) system [31]. Imaging catheters were fabricated in-house, comprising a single double-clad fiber (DCF; SM-9/105/125-20A, Nufern, East Granby, CT, USA) surrounded by a torque cable to allow for multimodal imaging in a small catheter. A graded index fiber is used to focus the beam and provide a lateral resolution of approximately 35 μm.

AF is generated with blue (445 nm) excitation light transmitted in the DCF core. Emission is collected in the cladding (>480 nm) and detected by a photomultiplier tube (PMT, H9433-201, Hamamatsu, Japan). Infrared light (1310 ± 50 nm; SSOCT-1310, Axsun Technologies Inc., Billerica, MA, USA) is transmitted and collected in the DCF core to provide OCT with an axial resolution of 7 μm in tissue. A custom DCF fiber optic rotary joint (Princetel, Hamilton Township, NJ, USA) and linear actuator allow for helical scan patterns to generate volumetric images of up to 16 cm in length. Light collected by the core and the cladding are separated using a double-clad fiber coupler (DCFC, DC530SEFA, Thorlabs, Newton, NJ, USA). Imaging catheters are housed in a 0.9 mm outer diameter window tube filled with sterile water for index matching, with pad-printed sheath markings at 5 cm and 7 cm along the length. Seven unique catheters were fabricated and used throughout the course of this study.

### 2.3. Image Collection

The fallopian tubes are resected from the specimen and imaging is conducted as soon as possible after arrival at the pathology department; the average time between sample arrival and time of imaging was 70 min.

Prior to imaging, fluorescent positive and negative standards are imaged for calibration purposes. First, an image of fingertips is taken to set the OCT reference and assess AFI quality. As a negative (dark) control, the imaging catheter is inserted into a 15 mL test tube of water covered in matte black aluminum foil, and an image is acquired. As a positive (bright) control, a 15 mL test tube of 0.98 μM fluorescein (selected for its similar intensity response to fingertips) is imaged; the catheter is positioned in the center of the test tube by two 3D-printed holders. After imaging the controls, the catheter is wiped down to remove remaining fluorescein.

**Figure 1:**
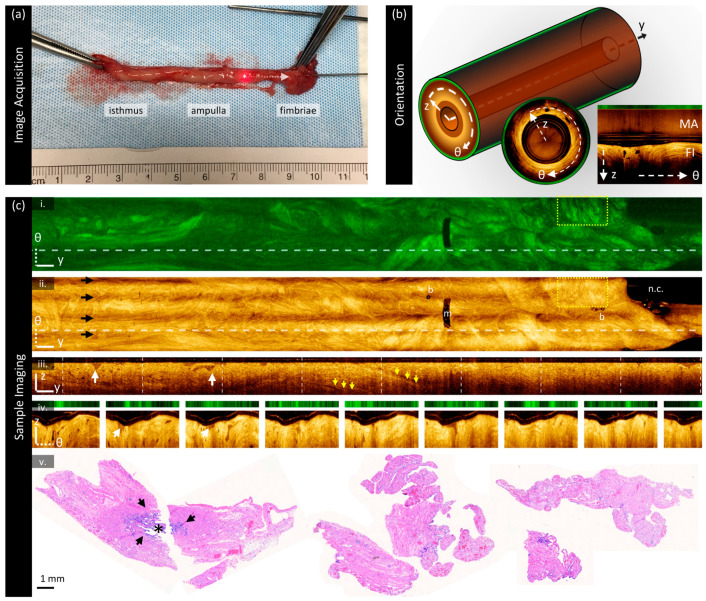
OCT orientation and sample imaging of a fallopian tube containing no lesion. (**a**) Endoscopic OCT is collected in a cylindrical volume (left); directions (‘z’, ‘θ’, ‘y’) are indicated. A sample cross-section (center) and an ‘unwrapped’ cross-section (right) are demonstrated. The unwrapped cross section includes the fundamental image (‘FI’) and multipath artifact (‘MA’). (**b**) Image acquisition methods. The imaging catheter is inserted into the infundibulum and positioned for image acquisition. The catheter is retracted in the distal direction (arrow) within the stationary window tube (tip indicated by ‘*’). (**c**) Sample imaging from a fallopian tube containing no lesions: (**i**) en face autofluorescence image; (**ii**) OCT mean en face projection; (**iii**) longitudinal section taken from the dashed line in the en face images; (**iv**) cross-sections taken from the dashed lines in the longitudinal section. (**v**) Representative histology sections. The yellow boxed region in panels **c**(**i**,**ii**) demonstrate regions where AFI and OCT capture visualize different features. The white arrows in panels **c**(**iii**,**iv**) highlight subsurface gaps in tissue. The yellow arrows in panels **c**(**iii**) indicate vessel-like subsurface structures. The black arrows in panel **c**(**v**) indicate the endosalpinx; the fallopian tube lumen is indicated with an asterisk (‘*’). All scale bars are 1 mm.

Image collection is shown in Figure 1a. Once the fallopian tube is resected, it is challenging to cannulate the isthmus as one would for in vivo falloposcopy. Instead, a metal grossing probe is used to identify the abdominal ostia within the fimbriated end to assist in cannulation (top right of Figure 1a). The imaging catheter is inserted into the fallopian tube until exit or until the sample is no longer catheterizable; the specimen is stabilized with forceps as needed. In Figure 1a, part of the imaging catheter is visible exiting the infundibulum on the right side of the photograph; the end of the imaging core is denoted with a (‘*’). A ruler is included in the photograph for co-localization of the imaged region. Once in position, the optical core of the imaging catheter is retracted within the stationary window tube in a helical scan pattern with a retraction speed of 1 mm/s (1792 A-lines per B-frame) to acquire volumes. Following imaging, the SEE-FIM protocol [22] is conducted: representative histological sections are taken approximately every 2 mm along the length of the sample and longitudinally at the fimbriae [66].

The three-dimensional orientation system is described in Figure 1b: y is the pullback dimension, θ is the angle around the pullback dimension, and z is the depth into the tissue. AFI is acquired en face (one intensity value collected per A-line). Most figures presented herein are en face (y-θ) mean intensity projections or unwrapped cross-sectional sections (θ-z). Images are presented proximal (left) to distal (right). The inset center and right images in Figure 1b show cross-sections (wrapped and unwrapped, respectively). In the unwrapped cross-section, multipath artifacts (‘MA’) are indicated at the top of the frame above the fundamental image (‘FI’) generated from the fundamental mode (LP01). Occasionally, there are additional multipath artifacts present below the fundamental image as well, representing higher-order modes coupled on both the forward and back-paths; these are often lower in intensity and may not be present in all images.

Sample imaging is shown in Figure 1c from a specimen containing no lesion or other abnormalities of note, cropped for viewing, focusing on the distal region. AFI (Figure 1c(i)) is acquired as one en face section (two-dimensional) and is presented on a scale from black (low fluorescence) to bright green (highly fluorescent). In this sample, some folding structures are visible with tendril-like darker regions interspersed with brighter areas.

OCT is presented in three views: mean en face projections (Figure 1c(ii)), longitudinal sections (Figure 1c(iii)) taken from the dashed line in the en face images, and cross-sections (Figure 1c(iv)), also referred to as B-scans, taken from the dashed lines in the longitudinal section. The cross-sections presented in this paper are ‘unwrapped’ for display. The multipath artifact is masked out in the cross-sections for viewing purposes.

OCT is presented on a colour scale from black (low intensity) to sepia (high intensity) corresponding to the magnitude of light returned from tissue. The en face OCT and AFI contain different features: for example, the yellow boxed region has many small, dark wrinkles in AFI but appears largely homogenous in OCT. Examining the longitudinal section or cross-sections reveals many small gaps in tissue, which we speculate are gaps between overlapping plicae (white arrows). There are also some longer structures (yellow arrows) visible in the longitudinal section, which may represent ducts or vasculature, as suggested by previous groups [40].

Several imaging artifacts are present in this volume, denoted in Figure 1c(ii). There are small bubbles (‘b’) within the window tube and a sheath marker (‘m’) which obscure the image. There is a region with no tissue contact on the right-hand side of the image (‘n.c.’). There are also four bands spanning the length of the en face volume (black arrows): this is a birefringence artifact generated by this OCT-AFI system which could be overcome in future studies through polarization-diverse detection [67].

Sample histology from this specimen is demonstrated in Figure 1c(v) through staining with hematoxylin and eosin (H&E). This specimen contains no lesion, and the sections are largely representative of the fimbriae (longitudinal sections). The leftmost two sections appear to be a distal cross-section: they show a lumen (‘*’) with folded plicae bordered by dark purple epithelium (black arrows). Proximal cross-sections (not pictured) contain a smaller lumen with simpler plicae structures.

### 2.4. Image Preparation

**Figure 2:**
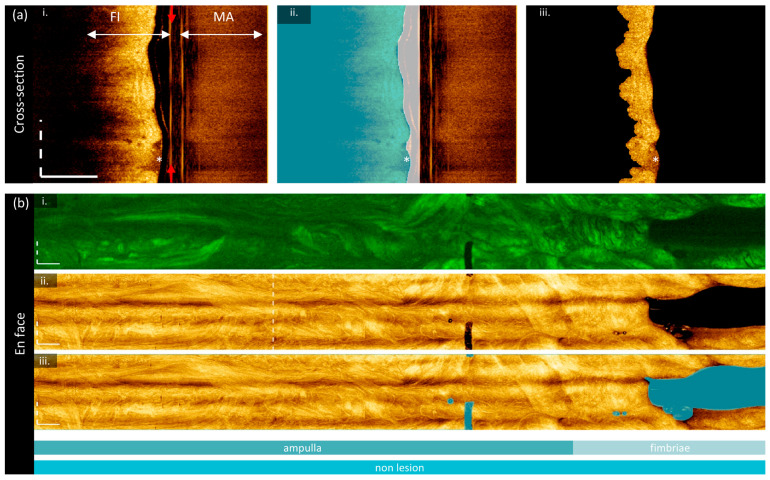
Image preparation for biomarker calculation. (**a**) Cross-sections are segmented to identify the luminal surface and imaging core (red arrow): (**i**) raw image demonstrating the multipath artifact (‘MA’) and fundamental image (‘FI’); (**ii**) segmentations: luminal region (blue) and optical core to luminal surface (white); (**iii**) final tissue segmentation excluding values < 6 dB above the noise floor. (**b**) En face segmentation demonstrating the original AFI (**i**) and OCT (**ii**), and the masked en face OCT (**iii**). The cross-section presented in (**a**) is taken from the dashed vertical line in (**ii**). Sites are co-localized below the en face image; the isthmus is cropped out for presentation and this sample contains no lesion. All scale bars are 1 mm.

Image preparation is required to ensure the correct region is measured before imaging biomarkers are calculated. Two groups of masks are generated: cross-sectional masks to identify the luminal surface (Figure 2a), and en face masks to remove imaging artifacts (Figure 2b). All image processing is conducted in MATLAB 2024a; deep learning predictions are generated using Python 3.10.0 with a PyTorch framework. All experiments were performed on a Windows 10 operating system, with Intel Core i7-12700K 3.60 GHz CPU, NVIDIA GeForce GTX 3080Ti GPU, and 32 GB of RAM.

#### 2.4.1. Cross-Sectional Lumen Segmentation

As the OCT volumes in this dataset comprise several thousand cross-sections, manual segmentation is intractable. The complexity of tissue and variety of presentation preclude classical segmentation approaches; thus, we use a previously developed deep learning segmentation tool [68].

Each cross-section is saved as an unwrapped .tif (Figure 2a(i)), resampled such that pixels at the window tube are 10 μm square (index of refraction of water is assumed in the A-line direction) and are smoothed with out-of-plane averaging, with 5 adjacent sections in each direction. These cross-sections are interpreted with a previously trained luminal segmentation deep learning network: a four-layer U-Net trained with 532 manually segmented cross-sections of endobronchial OCT from 39 lung transplant patients. This poses two domain transfer concerns: first, endobronchial tissue presents differently than fallopian tissue, and second, the OCT quality in OCT-AFI is lower and impeded by multipath artifacts. While future work may benefit from retraining a model to this use-case, we were able to generate sufficient quality segmentations through pre- and post-processing.

Before prediction, multipath artifacts (‘MA’, Figure 2a(i)) were masked to prevent spurious segmentations. The lowest point of the multipath artifact and an estimated lowest point of the fundamental image (‘FI’) were selected for each volume, and all values outside of the fundamental image region were set to zero. Cross-sections were tiled horizontally to prevent discontinuities in the azimuthal direction, zero-padded at the top of the frame to generate square tiles, and downsampled (192 × 192 pixel) for input into the network. Predictions were noisy and discontinuous, though they did identify the luminal surface. Post-processing (morphologic linking, combination with adjacent frames, thresholding) was required to generate a single continuous line from predictions. An example of the quality of segmentations produced by this approach is demonstrated in the blue region in Figure 2a(ii) (region filled below luminal segmentation). The result is imperfect but sufficient for our purposes; there are occasional gaps that are excluded from calculations, and this approach is biased toward including air or mucous (‘*’) rather than closely segmenting the plicae.

#### 2.4.2. Cross-Sectional Depth Segmentation

To allow for AFI calibration, the distance from the imaging probe to the tissue is required. A reflection from the outer diameter of the polyethelyne terephthalate (PET) tube attached to the optical core is used as a reference point (red arrows, Figure 2a(i)). This location is consistent across the volume, as the reference arm is not adjusted during acquisition; thus, it is calculated over the first 100 cross-sections to prevent outliers, and an average location is taken for the whole volume. First, all values below the luminal surface are masked. All A-lines in the cross-section are compressed into a sum projection along the azimuthal axis. The two most prominent peaks are identified, and the peak with a lower coordinate (outer diameter) is taken to be the desired location. This prevents erroneous identification of the higher order image (multipath artifact) from the optical core packaging reflection, which may appear similarly bright. The resulting region between the optical core and the luminal surface is presented using the white overlay in Figure 2a(ii).

This same procedure was applied to the positive and negative standards for AFI calibration; however, as those samples contain no tissue, the outer surface of the sheath was used instead of the luminal surface. The optical core location was identified first, without tissue masking, as the low-scattering fluorescein and water did not produce intensity peaks comparable to the plastic reflections. The outer surface of the sheath was identified by fitting a continuous line to a binary threshold mask of the image, set at 3 dB above the noise floor. Pixels within this mask which appeared more than 750 μm away from the optical core were set to zero to exclude the walls of the test tube.

#### 2.4.3. A-Line Truncation

Lastly, all values that are not at least 6 dB above the noise floor are excluded from the cross-section. The cross-sections presented in Figure 2a are oriented such that the A-line is increasing in index from right to left, where the left side of the frame is the deepest and the right side is the shallowest. If there is no multipath artifact present at the end of the A-line, the noise floor is calculated from 25 pixels (250 μm) at the end of the A-line (leftmost side of Figure 2a). Otherwise, the noise floor region is calculated from 25 pixels (250 μm) prior to (i.e., to the right of) the beginning of the multipath artifact. The selected region is smoothed with a 5-pixel Gaussian kernel, and the noise floor is taken to be the mean value of this region. The resulting region (‘visualized tissue’) for this cross-section is shown in Figure 2a(iii).

#### 2.4.4. En Face Segmentations

The mean en face projection of OCT was segmented manually with in-house annotation software [69]. The region of tissue to retain was segmented in one mask, and regions to remove (bubbles, sheath markers, other artifacts) were segmented in a second mask. A sample en face segmentation is visible in Figure 2a(ii), where the blue regions are to be masked out. This process was also conducted on the positive and negative controls for AFI calibration, and a region containing only air (background) in the tissue volume was selected for use as a background fluorescence value.

#### 2.4.5. Diagnostic and Regional Labels

We co-register imaging against the pathology report to provide diagnosis and region labels for each cross-section. These are best estimates of locations; diagnostic labels cover approximately 2 mm long cylindrical volumes, and regional labels are estimated retrospectively. A pathologist assesses each histologic section produced through the SEE-FIM protocol as a diagnostic gold standard. The coordinates of the ends of the specimen in the image are identified manually on the en face OCT and compared against the recorded length from the pathology report and photographs taken during imaging. If the imaged area is longer than that of the pathology report, we scale pathology lengths linearly to the imaged region. If the entire sample was not imaged (i.e., could not be cannulated fully), we use the pathology-measured lengths directly.

We measure the longest axis of the fimbriae from the histology slides and use the area from the end of the tissue to this length as the fimbriated region. The remainder of the specimen is divided into equally spaced regions by the number of cross-sections. Each region is assigned a diagnostic label (lesion/no lesion) according to its corresponding histologic section. The ampulla region is estimated to be 50% the length of the specimen starting at the beginning of the fimbriae region. Any other tissue is included in the isthmus region.

### 2.5. Biomarkers

**Figure 3:**
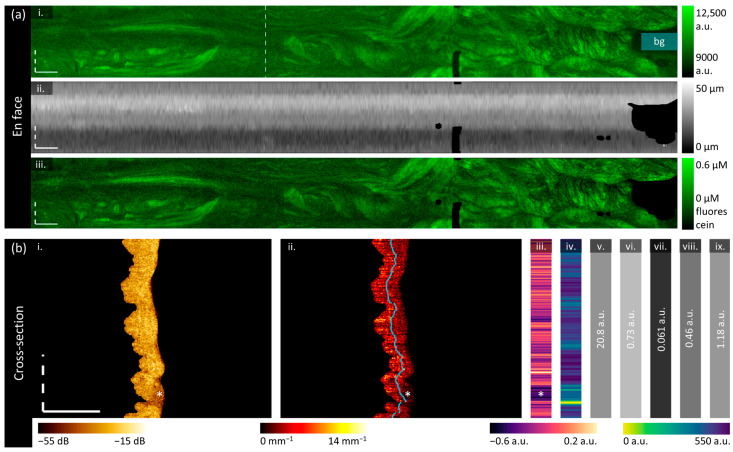
Biomarker calculation. (**a**) Calculation of en face functional biomarkers: (**i**) the original AFI with the background region (‘bg’) manually identified; (**ii**) the depth map constructed as the distance from the optical core reflection to the tissue surface for each A-line; (**iii**) the final calibrated AFI in units of fluorescein. (**b**) Calculation of cross-sectional (attenuation and texture) biomarkers from the dashed line in panel (**a**(**i**)): (**i**) the segmented OCT section; (**ii**) the depth-resolved attenuation coefficient cross-section with the regions of superficial and deep attenuation coefficient divided by the blue line; two-dimensional feature that results in one value per A-line: (**iii**) stratification and (**iv**) speckle contrast; and one-dimensional GLCM features: (**v**) contrast, (**vi**) correlation, (**vii**) energy, (**viii**) homogeneity, and (**ix**) Shannon entropy. All scale bars are 1 mm.

After image preparation, each cross-section has a mask for the tissue region, the depth between the imaging core and tissue, and labels for diagnostic state and region. Eleven biomarkers are selected for investigation, as described in Table 1 and demonstrated in Figure 3. This includes features related to functional characteristics (AFI), optical attenuation (OCT), and texture (OCT). After calculation, each measurement is rescaled such that all pixels are 10 μm square and reinterpreted as a two-dimensional en face view (through mean projection for three-dimensional features) for comparison against diagnostic and region labels. The median value of each feature over the region of interest is used as the measurement for later statistical analysis.

#### 2.5.1. Functional Features

Raw autofluorescence intensity is subject to variations in the OCT-AFI system, including but not limited to laser intensity, detector sensitivity, changes in the coupling fluid within the fiber optic rotary joint, and the coupling ability of differences between imaging catheters. To improve the comparability of measurements presented in this study, a calibration approach was implemented to rescale intensity values with respect to a positive and negative standard, the background intensity within the volume containing tissue, and the depth from the optical core, as demonstrated in Figure 3.

Using the optical core reflection and sheath or tissue segmentations, an en face depth map is constructed for the tissue volume and the two standard volumes (tissue depth map shown in Figure 3a(ii)). A simple model is fit to estimate intensity as a function of depth for both standards. In the tissue volume, the mean background intensity is calculated over the manually selected region of air at the exit of the fallopian tube (‘bg’ in Figure 3a(i)). For each pixel in the original en face AFI (Figure 3a(i)), the calibrated intensity (Figure 3a(iii)) is taken to be the intensity of the raw tissue pixel with the mean background intensity subtracted off, divided by the difference between the bright and dark calibration curves at the corresponding depth of that pixel, multiplied by the fluorescein concentration of the positive standard. This approach allows AFI to be reported in terms of μM of fluorescein.

We anticipate that calibrated AFI will provide an improved measurement of tissue fluorescence and will minimize system impacts. We expect that areas of lesion will appear as regions of low fluorescence compared to surrounding tissue.

#### 2.5.2. Attenuation Features

The optical attenuation coefficient (μ) describes the scattering and absorptive properties of tissue by characterizing the exponential decay of light in the A-line direction. It can be estimated in a depth-resolved manner from OCT data, producing a new three-dimensional volume of estimated attenuation at each voxel [72]. This is more quantitative than OCT intensity alone, and it compensates for variations due to the power source, catheter quality, reference position, and/or user handling. The tissue-specific contrast provided by the optical attenuation coefficient has been explored in a variety of cancer detection applications [44]. While attenuation coefficient values for tubo-ovarian cancers measured with falloposcopy have not been reported, measurements from the fallopian tube exterior with a micromotor catheter have demonstrated a decrease in the attenuation coefficient in cancer [40].

We implement the algorithm described by Liu et al. [70] over the segmented tissue region, resulting in the cross-section demonstrated in Figure 3b(ii). We assess the overall attenuation coefficient (mean projection over the entire depth of tissue), the superficial attenuation coefficient (mean projection over the upper 50% of tissue depth, above the blue line in Figure 3b(ii)), and deep attenuation coefficient (mean projection over the lower 50%). Generally, we visualize <500 μm of tissue, and so we anticipate that the superficial region will contain endosalpinx and some myosalpinx, whereas the deep region will contain primarily myosalpinx, though this is complicated due to the folded nature of the plicae. Examining the example in Figure 3b(ii), there appears to be subtle stratification with a layer of lower attenuation coefficients close to the luminal surface underlain with a layer of higher attenuation values; calculating the mean projection of the attenuation coefficient over individual depth regions will allow us to examine these changes.

We calculate a ratiometric stratification biomarker to further examine the differences between the superficial and deep regions (Figure 3b(iii)). This is calculated through the difference between the superficial and deep projections divided by their sum. Negative values correspond to a higher-value deep attenuation coefficient, and positive values correspond to a higher-value superficial attenuation coefficient. From the example cross-section, we see that generally, the attenuation coefficient is lower in the superficial region, and the region containing mucous rather than tissue (‘*’) has very low attenuation coefficient values in the superficial region. We anticipate that carcinoma may appear as a loss of stratification, wherein the superficial and deep regions become homogenous with invasion.

#### 2.5.3. Speckle Features

Speckle is inherent to low-coherence imaging methods and may contain sub-resolution characteristics [49]. We estimate the speckle distribution by fitting the intensity data (no out-of-plane averaging with other cross-sections) to a gamma distribution as described by Lindenmaier et al. [50]. To achieve an estimation of this distribution per A-line while ensuring enough datapoints for a reasonable fit, we combine 5 adjacent A-lines in each direction and include the entire region of tissue depth. A-lines with a tissue depth of less than 10 pixels are excluded. The mean of the gamma distribution is described by α/β, where α is the shape parameter of the gamma distribution and β is the scale parameter. We use the mean of the gamma distribution as our biomarker, which is presented in Figure 3b(iv). In a cervical cancer mouse xenograft study, a lower α/β value was found in tumors when compared to normal tissue [50] even when changes were not visually distinguishable in OCT, so we anticipate this feature may appear similarly in our application.

#### 2.5.4. Gray Level Co-Occurrence Matrix (GLCM) Features

GLCM features have been proposed in many medical imaging applications to describe textural changes [46,73]. We calculate these features on the log-transformed intensity data without out-of-plane averaging but after resampling each pixel to be 10 μm square.

First, the 5th and 95th quantiles of the entire volume are calculated as the minima and maxima for normalization. OCT data are masked to only include the tissue region, normalized to [0, 1] using the identified minima and maxima, and binned to 32 gray levels. A GLCM is generated using the MATLAB graycomatrix function with a 1-pixel shift in the azimuthal (fast axis) direction. Five Haralick features are calculated on the GLCM as described in Table 1, resulting in one value per cross-section (Figure 3b(v–ix)).

This study does not seek to report all possible GLCM features nor optimize normalization methods, binning, or directionality approaches. We elect to limit the number of calculated features to common radiomic feature descriptions with clear definitions of texture behaviors. Previous work has demonstrated that textural features in combination with a classifier can distinguish tumors in transgenic mouse models of ovarian cancer [47]. This study found energy, correlation, contrast, and homogeneity to be the most statistically significant for differentiating treatment groups in two dimensions, and Shannon entropy demonstrated the best ability to discriminate groups in three dimensions. While we are implementing this in a two-dimensional approach in human fallopian tissue, we anticipate that we may be able to detect similar changes with the same approach.

### 2.6. Statistical Analysis

Statistical analysis is conducted in TIBCO Statistica 14. A single median value is used for each biomarker over the desired region in each volume for statistical analysis. The cross-sectional tissue masks and en face artifact masks described in Section 2.4 are used to exclude measurements in regions with artifacts, bubbles, low-signal, or non-tissue contact.

As our sample size is small and limited when it comes to volumes containing lesions of interest, we do not correct for multiple comparisons and are not able to control for confounding covariates. This is a hypothesis-generating study intended to provide future direction and to examine potential diagnostic features; additional study is required to confirm their utility. We have selected a significance level of *p* < 0.05 for all tests.

We explore several statistical questions described in Table 2 that compare the measured biomarkers to various dependent variables. The Shapiro–Wilk W test [74] is used to test biomarkers for normality and assess which features require parametric or non-parametric tests. For paired tests, missing data are excluded in a pairwise fashion. For unpaired tests, assumptions of homogeneity of variances are assessed with Levene’s test [75], and the presence of outliers is assessed with Grubb’s test [76].

## 3. Results

### 3.1. Dataset

A novel dataset of volumetric structural (OCT) and functional (AFI) imaging of ex vivo fallopian tubes with corresponding histopathology was collected. This includes samples from 28 unique patients as described in Table 3, with 7/28 specimens containing cancerous lesions (LGSOC, HGSOC, carcinoid). Only one specimen (one of the HGSOC cases) contains STIC.

In addition to cancerous lesions of interest, 2/28 specimens contain endometriosis lesions. We include and present these specimens as they demonstrate striking imaging features that are not similar to carcinoma which may add complexity to image interpretation.

We describe samples containing no lesion within the fallopian tube as ‘non-lesion’ or ‘non-cancerous’ specimens. This is to reflect that they are recruited from patients who consented to the Gynecologic Tissue Bank who have undergone salpingectomy, and while the fallopian tube may contain no lesions, there may be a cancer present in other parts of the gynecologic tract.

Five patients were imaged bilaterally (including both left and right fallopian tubes), but only one fallopian tube per patient was included in the subsequent analyses. If one side contained a lesion and the other did not, the former was included in the dataset. If both sides presented with the same diagnostic status, the specimen to include was selected with a random number generator. The bilateral imaging is summarized in Table 4 and is used for the reproducibility analyses described in Section 3.4.

### 3.2. Sample Imaging

**Figure 4:**
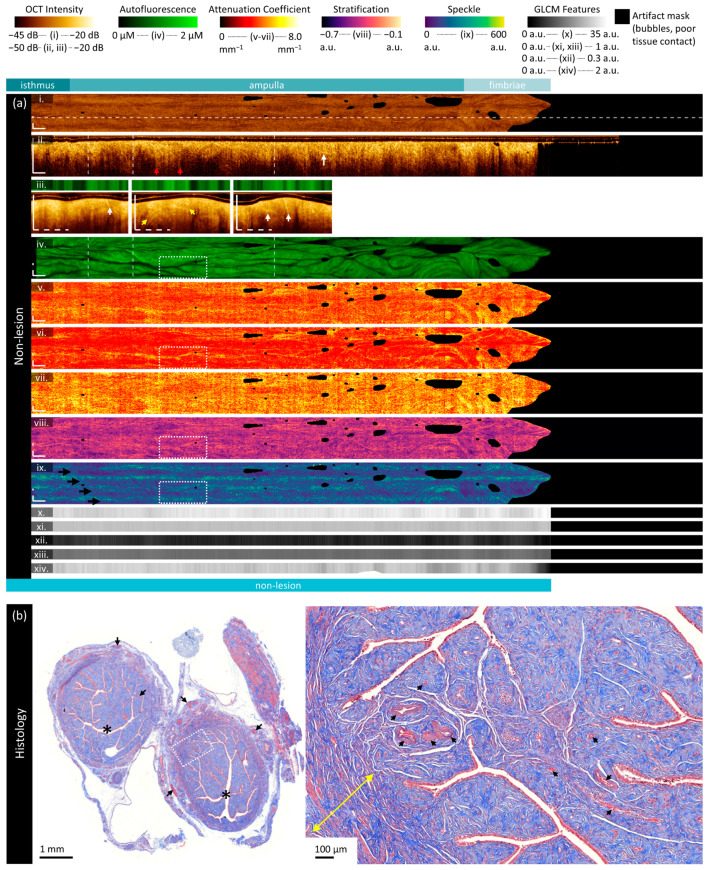
Sample imaging of a fallopian tube containing no lesion. Panel (**a**) demonstrates the biomarkers: (**i**) mean OCT en face projection; (**ii**) longitudinal OCT section from the dashed line in panel (**i**); and (**iii**) three sample OCT cross-sections from dashed lines in the longitudinal section demonstrated in wrapped and unwrapped views alongside co-registered AFI. Longitudinal and depth sections are cropped to the region containing the fundamental image. The biomarkers are presented below: (**iv**) autofluorescence; (**v**) overall attenuation coefficient; (**vi**) superficial attenuation coefficient; (**vii**) deep attenuation coefficient; (**viii**) stratification; (**ix**) speckle distribution; (**x**) GLCM contrast; (**xi**) GLCM correlation; (**xii**) GLCM energy; (**xiii**) GLCM homogeneity; and (**xiv**) GLCM Shannon entropy. Arrows and boxes indicate features described in the text: OCT multiple scattering ‘tails’ (red arrows); folded epithelium or vessel-like structures (white arrows and white boxed region); tissue stratification (yellow arrows); and birefringence banding artifacts (black arrows). Panel (**b**) demonstrates representative histology (Masson’s Trichrome); the right panel is inset from the boxed region on the left panel. The lumen is identified by an asterisk (‘*’), vessels with black arrows, and the myometrium with yellow arrows. All scale bars are 1 mm unless otherwise noted.

**Figure 5:**
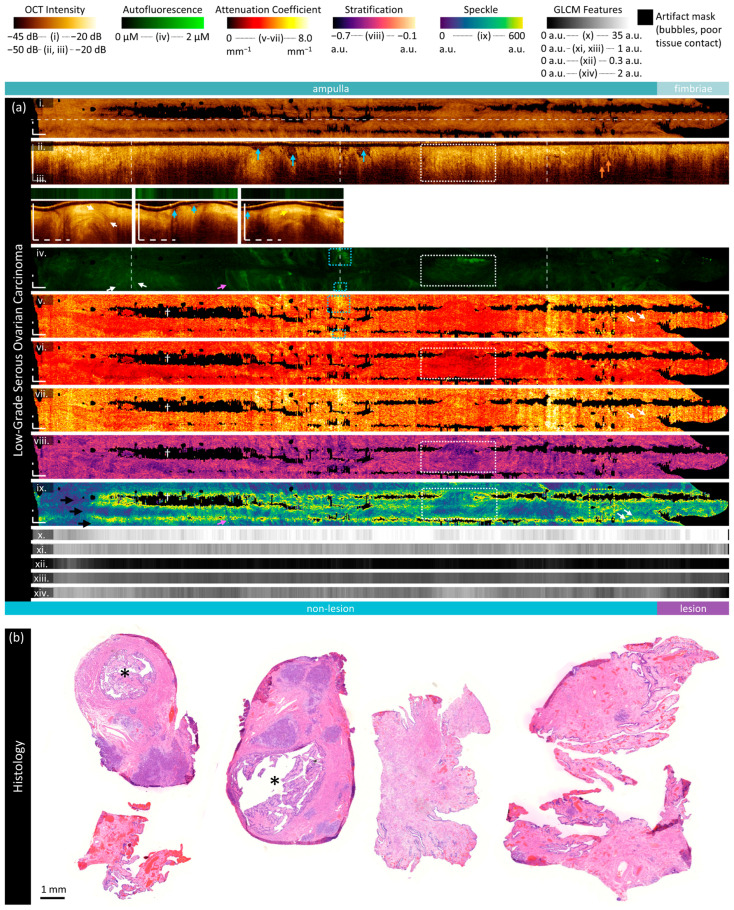
Sample imaging of a fallopian tube containing LGSOC. Panel (**a**) demonstrates the biomarkers: (**i**) mean OCT en face projection; (**ii**) longitudinal OCT section from the dashed line in panel (**i**); and (**iii**) three sample OCT cross-sections from dashed lines in the longitudinal section demonstrated in wrapped and unwrapped views alongside co-registered AFI. Longitudinal and depth sections are cropped to the region containing the fundamental image. The biomarkers are presented below: (**iv**) autofluorescence; (**v**) overall attenuation coefficient; (**vi**) superficial attenuation coefficient; (**vii**) deep attenuation coefficient; (**viii**) stratification; (**ix**) speckle distribution; (**x**) GLCM contrast; (**xi**) GLCM correlation; (**xii**) GLCM energy; (**xiii**) GLCM homogeneity; and (**xiv**) GLCM Shannon entropy. Arrows and boxes indicate features described in the text: regions where there is no contact between the tissue and catheter (blue arrows); folded epithelium or vessel-like structures (white arrows and white boxed region); regions with tissue stratification (yellow arrows and blue boxes); subsurface voids or edema (orange arrows); regions of non-calculation due to low signal (†); and birefringence artifacts (black arrows). Panel (**b**) demonstrates representative histology; the lumen is identified by an asterisk (‘*’). All scale bars are 1 mm.

**Figure 6:**
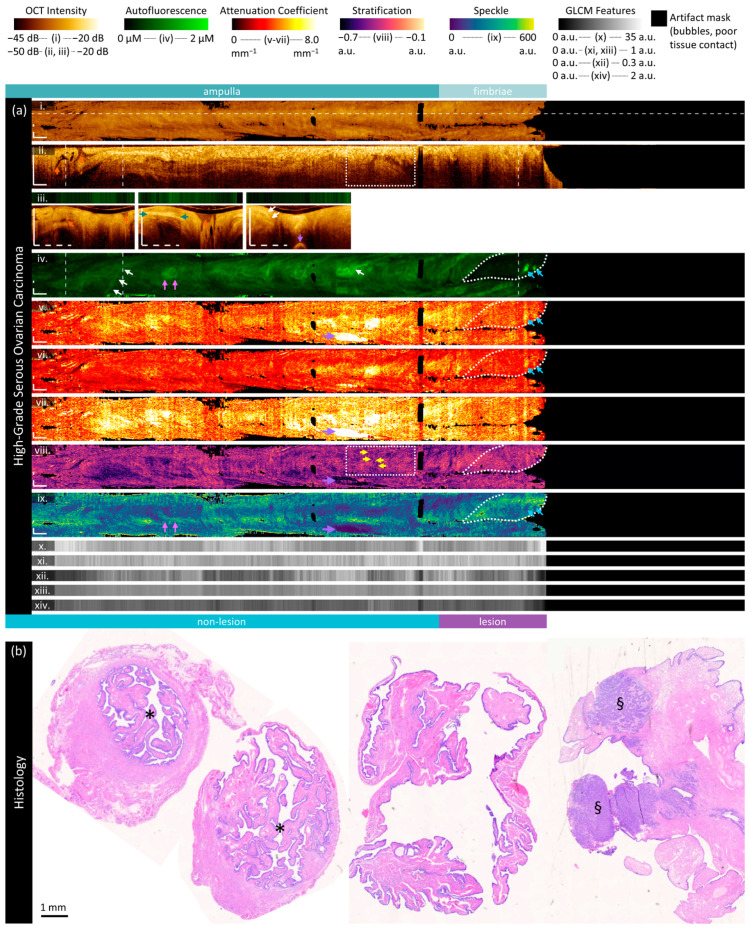
Sample imaging of a fallopian tube containing HGSOC. Panel (**a**) demonstrates the biomarkers: (**i**) mean OCT en face projection; (**ii**) longitudinal OCT section from the dashed line in panel (**i**); and (**iii**) three sample OCT cross-sections from dashed lines in the longitudinal section demonstrated in wrapped and unwrapped views alongside co-registered AFI. Longitudinal and depth sections are cropped to the region containing the fundamental image. The biomarkers are presented below: (**iv**) autofluorescence; (**v**) overall attenuation coefficient; (**vi**) superficial attenuation coefficient; (**vii**) deep attenuation coefficient; (**viii**) stratification; (**ix**) speckle distribution; (**x**) GLCM contrast; (**xi**) GLCM correlation; (**xii**) GLCM energy; (**xiii**) GLCM homogeneity; and (**xiv**) GLCM Shannon entropy. Arrows and boxes indicate features described in the text: lesion (white dashed region); regions of high fluorescence response (blue and pink arrows); vessel-like structures (white arrows); folding structures (orange arrows); regions with tissue stratification (green arrows) and subsurface gaps (boxed region and yellow arrows); and a reflection from the forceps exterior to the specimen (purple arrows). Panel (**b**) demonstrates representative histology; the lumen is identified by an asterisk (‘*’) and the lesion with ‘§’. All scale bars are 1 mm.

**Figure 7:**
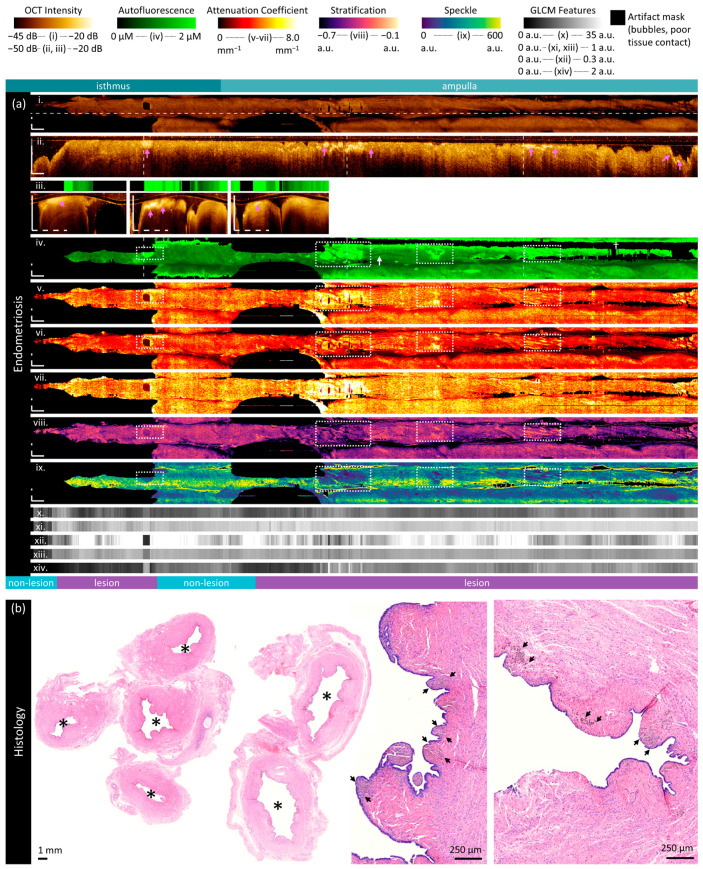
Sample imaging of a fallopian tube containing endometriosis. Panel (**a**) demonstrates the biomarkers: (**i**) mean OCT en face projection; (**ii**) longitudinal OCT section from the dashed line in panel (**i**); and (**iii**) three sample OCT cross-sections from dashed lines in the longitudinal section demonstrated in wrapped and unwrapped views alongside co-registered AFI. Longitudinal and depth sections are cropped to the region containing the fundamental image. The biomarkers are presented below: (**iv**) autofluorescence; (**v**) overall attenuation coefficient; (**vi**) superficial attenuation coefficient; (**vii**) deep attenuation coefficient; (**viii**) stratification; (**ix**) speckle distribution; (**x**) GLCM contrast; (**xi**) GLCM correlation; (**xii**) GLCM energy; (**xiii**) GLCM homogeneity; and (**xiv**) GLCM Shannon entropy. Arrows and boxes indicate features described in the text: hemosiderin deposits (white dashed boxes and pink arrows) and vessel-like structures (white arrow). Panel (**b**) demonstrates representative histology; the lumen is identified by an asterisk (‘*’) and hemosiderin deposits with black arrows. All scale bars are 1 mm.

We present sample imaging and biomarkers in Figure 4, Figure 5, Figure 6 and Figure 7, including examples of a non-cancerous fallopian tube, LGSOC, HGSOC, and endometriosis. Each of the subsequent figures follows the same presentation: (a) visual demonstration of the biomarkers and (b) representative histology. Measurements of the biomarkers for each sample are included in the Supplementary Materials (Figure S1). Colour bars are consistent across the presented figures for comparison; scale bars are 1 mm unless otherwise noted. Volumes are cropped or zero-padded on the right-hand side to allow for presentation with consistent scaling.

#### 3.2.1. Non-Lesion Specimen

This specimen (Figure 4) is from a 78-year-old patient and contains no lesion. The isthmus, ampulla, and fimbriae are included in the imaged region.

The mean en face projection of OCT (Figure 4a(i)) is largely homogenous, though examination of the longitudinal OCT section (Figure 4a(ii)) reveals textural changes along the length of the fallopian tube. The visualized depth of tissue varies substantially in the longitudinal direction, with some regions having long ‘tails’ (red arrows). This suggests multiple scattering in some (but not all) regions. Similar ‘icicle-like’ structures have been proposed to be suggestive of neoplastic infiltration in oral OCT [83].

The OCT cross-sections (Figure 4a(iii)) contain branching regions of increased backscattering (white arrows) extending outwards from the luminal surface. These are also visible in the longitudinal section (white arrows) but are more subtle. We speculate that these could be folded epithelium or vasculature. Inserting the imaging catheter (0.9 mm diameter) into the lumen (‘*’, Figure 4b) will have distended and distorted the plicae, which could result in overlapping folds of epithelium deeper within the tissue. Alternatively, while larger vasculature is present in the serosa (black arrows, Figure 4b left), there are smaller vessels present near the lumen (black arrows, Figure 4b right).

There are subsurface bands of increased intensity in the central OCT cross-section (yellow arrows), which may indicate a transition to a different region of tissue. This may be a transition between folded and overlapping plicae or may capture muscle fibers of varying directions in the myosalpinx (yellow arrows, Figure 4b right inset).

The autofluorescence image (Figure 4a(iv)) demonstrates dark tendril-like features threaded throughout the volume. These correspond to the bright branching structures in OCT, further supporting speculation that these structures are vasculature or epithelium. We have previously reported brightly fluorescent vasculature networks in endobronchial OCT-AFI [30]; however, we have occasionally visualized dark vasculature networks, particularly in endobronchial imaging of lung transplant patients and when vasculature is near the luminal surface. As the predominant fluorophores captured in blue-light AFI are from collagen in the extracellular matrix, tissue with increased epithelial depth often appears low-fluorescence. In general, there are no regions with particularly high or low autofluorescence that are uncorrelated to distance or branching structures in OCT.

Optical attenuation is higher in the deep region (median 4.00 mm^−1^; Figure 4a(vii)) than in the superficial region (median 3.03 mm^−1^, Figure 4a(v)). This generally holds over all regions, though there is a slight increase in the attenuation coefficient in the fimbriae. An examination of the deep attenuation coefficient and the stratification metric (Figure 4a(viii)) reveals the same vessel-like or folded epithelial structures (white box) as in an increase in optical attenuation contribution from the superficial region (Figure 4a(vi)).

The speckle metric (Figure 4a(ix)) is affected by the birefringence artifact, which appears as four longitudinal bands (black arrows) of increased value. While this artifact is minimal in this sample and is largely not distinguishable in the other biomarkers, it does impact the speckle contributions. The vessel-like or folded epithelial structures (white box) appear as a lower mean speckle distribution.

GLCM texture features (Figure 4a(x–xiv)) appear to capture small variations along the length of the specimen, with very tight error bars for most features (see Supplementary Materials, Figure S1a). The exception to this is Shannon entropy, which is lower and has a wider variance in the fimbriae than in the other regions.

#### 3.2.2. Low-Grade Serous Ovarian Carcinoma

The specimen in Figure 5 is from a 69-year-old patient and contains low-grade serous ovarian carcinoma throughout the fimbriae. The isthmus is not included in the imaged region. Sample histology (Figure 5b) includes two cross-sections with the lumen marked with ‘*’ and several sections of fimbriae.

The visualization of subtle features in the attenuation, stratification, and speckle is impeded by the birefringence artifact in this volume. The birefringence artifact results in regions of non-calculation (black blocky regions, ‘†’) along the artifact bands (black arrows, Figure 5a(ix)). The fourth dark band is not easy to visualize in this presentation, as it is split around the top and bottom of the en face frame. In the longitudinal OCT, these artifacts appear as a reduction in the recorded backscattering.

The OCT longitudinal sections and cross-sections (Figure 5a(ii,iii)) demonstrate some similar features to the non-cancerous specimen: the visualized depth varies over the length of the volume, vessel-like or folded epithelial structures are present (white arrows), and bands of high-intensity scattering (yellow arrows) appear to differentiate regions of tissue layers into a low-intensity surface underlain by a layer of increased intensity. There are regions of non-contact (blue arrows) that show plicae, as this specimen is not entirely distended by the insertion of the imaging catheter.

Overall, the autofluorescence (Figure 5a(iv)) intensity is lower in this specimen (median intensity of 0.26 μM) than in the non-cancerous specimen (median 0.85 μM). Quantitative measurements for these sample cases are provided in the Supplementary Figures (Figure S1b). Within this sample, the fimbriae (where the lesion is present) has lower autofluorescence (median 0.16 μM) than the rest of the sample (median 0.27 μM).

The vessel-like or folded epithelial structures (white arrows in all panels) are much less prominent than in the non-lesion volume. The structure indicated in the first OCT cross-section (white arrows, Figure 5a(iii)) corresponds to a darkened region in the AFI; however, there are brightly fluorescent vessel-like structures throughout the volume (white arrows, Figure 5a(iv)). These correspond to branching structures close to the luminal surface in OCT cross-sections (not pictured). We speculate that brightly fluorescent structures are more likely to be vessels than folded epithelium.

The regions with tissue layering visible in the cross-sections (yellow arrows, Figure 5a(iii)) generally correlate with regions that have increased autofluorescence (blue boxes) and a high overall attenuation coefficient.

There are several short, circular voids present below the tissue surface (orange arrows) which appear similar to what has previously been reported as edema [39]. These features appear more frequently in regions with inconsistent tissue contact and appear to correlate with regions of increased overall attenuation (yellow box, Figure 5a(v)) and increased mean speckle distribution (yellow box, Figure 5a(ix)).

The speckle features (Figure 5a(ix)) are substantially obscured by the birefringence artifact, appearing as regions of increased mean speckle distribution. A few vessel-like features appear as lower mean speckle distribution, as identified by AFI (pink arrows). Additionally, some regions of decreased superficial attenuation, corresponding to increased stratification difference and increased autofluorescence, appear as lower mean speckle distribution (white box). This region appears to contain tissue layering similar to the third cross-section (not pictured).

As in the non-lesion volume, the GLCM Shannon entropy (Figure 5a(xiv)) is lower in the fimbriae and the area of lesion (median of 0.57 compared to 0.97). Regions of increased Shannon entropy appear to map to regions of decreased mean speckle distribution. The GLCM contrast is higher in the fimbriae and the area of lesion (Figure 5a(x); 34.6 compared to 32.1). The energy and homogeneity features are similar across all regions.

#### 3.2.3. High-Grade Serous Ovarian Carcinoma

This specimen (Figure 6) is from a 77-year-old patient and contains high-grade serous ovarian carcinoma throughout the fimbriae. Sample cross-sections and fimbriae are demonstrated in Figure 6b; the lumen is marked with a ‘*’ and the lesion with ‘§’.

There are striking features indicated by the purple arrows in the attenuation coefficient and stratification features; however, these are artifacts generated by reflections off of the metal pathology forceps used to position the specimen. An example of this is shown in the bright reflection in the third cross-section (purple arrows, Figure 6a(iii)).

The longitudinal OCT (Figure 6a(ii)) and first cross-section (Figure 6a(iii)) contain prominent folding structures (orange arrows) in the ampulla, as seen in the LGSOC specimen. These folds are well differentiated in the cross-section and demonstrate that in some regions, most of the viewing range may comprise overlapping plicae including both endosalpinx and myosalpinx. Immediately distal to this region in the longitudinal OCT, there is a sharp transition to a layered morphology: between the green arrows, at least four distinct layers are visualized, alternating between high-, very high-, high-, and low-intensity OCT. Three of these layers continue until the sheath marker or fimbriae region. The distal-most part of this transition is disordered and can be visualized as small dark dots in the stratification image (yellow arrows, Figure 6a(viii)). We speculate that the high scattering regions are endosalpinx, and the lowermost region is the transition to myosalpinx. The brightest layer does not appear to map to vascular structures in AFI, instead appearing as a low attenuation region with low mean scattering distribution.

AFI presents similarly to that of the LGSOC in terms of content, but with a higher median intensity. There are, again, a few vessel-like structures (white arrows) present in OCT cross-sections and AFI (Figure 6a(iv)). There is a circular high-fluorescence structure (pink arrows) which corresponds to a region of decreased mean speckle distribution. Examining the longitudinal OCT, this appears to be from the region with the thickest high-intensity (‘endosalpinx-like’) layer, which also has a gap in tissue structure below it.

Most importantly, the HGSOC can be visualized as a loss of fluorescence intensity in the white circumscribed region of the fimbriae. From the third OCT cross-section (Figure 6a(iii)), it is clear that this region contains tissue, but it is more homogeneous than other regions. The attenuation coefficients are lower, particularly the deep attenuation coefficient. There is less stratification (biomarker closer to zero) and a slight visual increase in speckle distribution. Within the area of lesion, there is increased scattering corresponding to the two highly fluorescent foci (blue arrows) with a high attenuation coefficient and low mean speckle distribution. A small vessel-like structure is visible in the OCT cross-section (white arrows), which can also be visualized in the speckle distribution (Figure 6a(ix)).

Speckle and GLCM features are generally consistent in this volume across all regions of interest. GLCM contrast (Figure 6a(x)) is slightly lowered in the fimbriae/lesion area. Notably, the measurements calculated in Figure S1c include the entire region denoted as the area of lesion below panel a; the region of distinct low fluorescence is only about 50% of this region, which may reduce the measured differences between areas of lesion and non-lesion.

#### 3.2.4. Endometriosis Specimen

The specimen in Figure 7 is from an 80-year-old patient with hydrosalpinx, and it contains hemosiderin deposits from endometriosis lesions. Figure 7a has been cropped for viewing; the entire volume includes the fimbriae. Figure 7b includes sample cross-sections with the lumen marked with ‘*’. Hydrosalpinx and inflammation has resulted in a much larger lumen with less complex folded plicae. Hemosiderin deposits are near the epithelial surface and appear as greyish deposits indicated by black arrows.

In OCT, plicae appear bulbous, and there is poorer tissue contact with the imaging probe. The tissue appears much more homogenous in the longitudinal OCT section (Figure 7a(ii)); layers are not easily distinguished, and the depth of visualized tissue is much more consistent over the length of the volume. Vessel-like structures are less visible but still present (white arrows).

In general, there are less birefringence artifacts in this specimen, but it is impacted by poor tissue contact with the imaging catheter. There are some regions (‘†’) where AFI calibration is not performed as the depth mask is more than 700 μm from the imaging core. Overall, the autofluorescence of this entire specimen is much higher than the previous samples (median 1.34 μM). We speculate this is related to the hydrosalpinx, as the AFI is predominantly driven by extracellular matrix behaviour in the region close to the surface of the imaging probe. Inflammatory and/or fibrotic processes during the distension of the fallopian tube may lead to an increase in tissue autofluorescence.

Hemosiderin fluoresces at 450 nm [84], which results in highly fluorescent regions in AFI (white boxes, Figure 7a(vi)). These can be mapped to high-intensity OCT just below the luminal surface (pink arrows, Figure 7a(ii,iii)), high superficial attenuation (white boxes, Figure 7a(vi)), positive stratification values (white boxes, Figure 7a(vi)), and low mean speckle distribution (white boxes, Figure 7a(vi)). They also appear dissimilar from their surroundings in all GLCM metrics (Figure 7a(x–xiv)). However, although these lesions are within the area labelled as ‘lesion’, indicating good co-registration in this volume, they only represent a small portion of the overall lesion area and thus are largely indistinguishable in the quantitative measurements (Figure S1d).

There are a few site-specific differences in the quantitative measurements (Figure S1d). The GLCM Shannon entropy of the isthmus is lower than that of the ampulla or the fimbriae, which are comparable. The GLCM contrast and autofluorescence increase over the length of the specimen from the isthmus through to the fimbriae. The attenuation coefficients in this specimen are slightly lower in the fimbriae than in surrounding regions.

### 3.3. Quantitative Comparison of Biomarkers and Disease State

**Figure 8:**
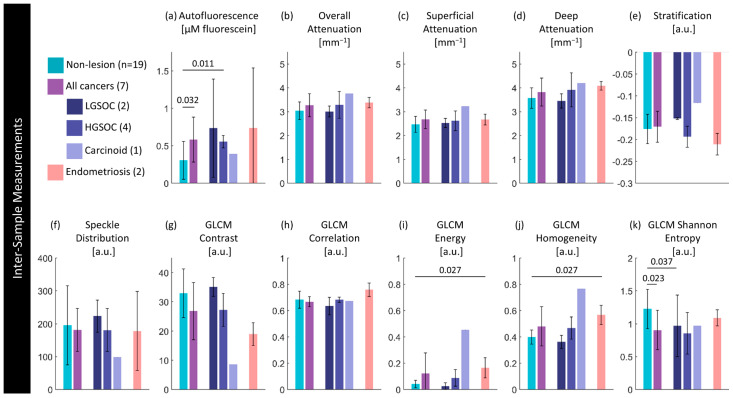
Inter-sample measurements of each feature per diagnosis. Values are calculated using the median measurement for each biomarker per volume. The height of bars represents the mean value of specimens in that group; error bars are standard deviation. *p*-Values for significant differences (*p* < 0.05) between disease states are indicated.

Figure 8 compares the median value for entire specimens against disease state; detailed values are provided in the Supplementary Table S1. This captures a significant increase (*p* < 0.05) in autofluorescence in specimens containing a cancerous lesion when compared to non-lesions, particularly in LGSOC (*p* < 0.05). We hypothesize that, as in the sample presented in Figure 7, this may be capturing inflammatory or fibrotic changes throughout the specimen. There are also some significant differences in texture features between specimens containing no lesion and specimens containing endometriosis lesions. There is a significant increase in GLCM energy (*p* < 0.05) and homogeneity (*p* < 0.05). There are other non-significant trends: cancerous lesions have a slight increase in attenuation in all regions; and speckle distribution appears to present differently for each cancer type, though this is substantially limited by sample size.

**Figure 9:**
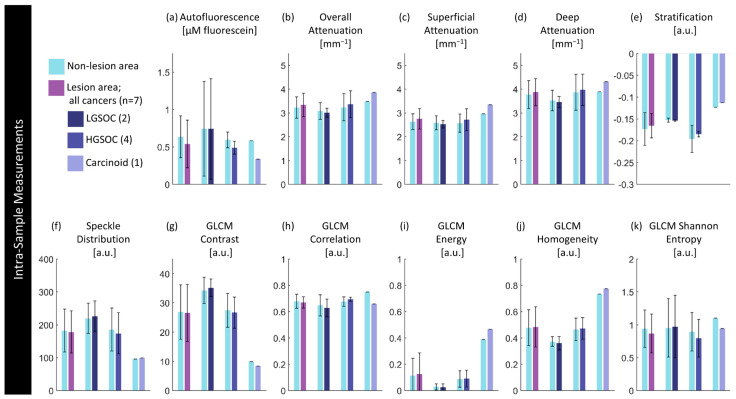
Intra-sample measurements of each feature per diagnosis. Values are calculated using the median measurement for each biomarker per region for specimens containing a lesion of interest. The height of bars represents the mean value of specimens in that group; error bars are standard deviation. There are no significant differences (*p* < 0.05) between paired lesion/non-lesion groups.

Figure 9 quantitatively compares biomarkers against disease state within specimens containing a lesion. Detailed values are provided in the Supplementary Table S2.

There are no significant differences between groups; however, these results are limited in terms of the precision of our ability to co-register with histology. This presents trends demonstrating that lesions appear to have lower intensity autofluorescence in the area of lesion compared to the non-lesion area, which follows from our visual inspections in Section 3.2. Optical attenuation is slightly increased in areas of HGSOC or carcinoid lesion compared to in non-lesion areas in those specimens. Stratification and textural differences are minimal.

### 3.4. Regional Assessment, Demographic Relations, and Other Potential Confounders

**Figure 10:**
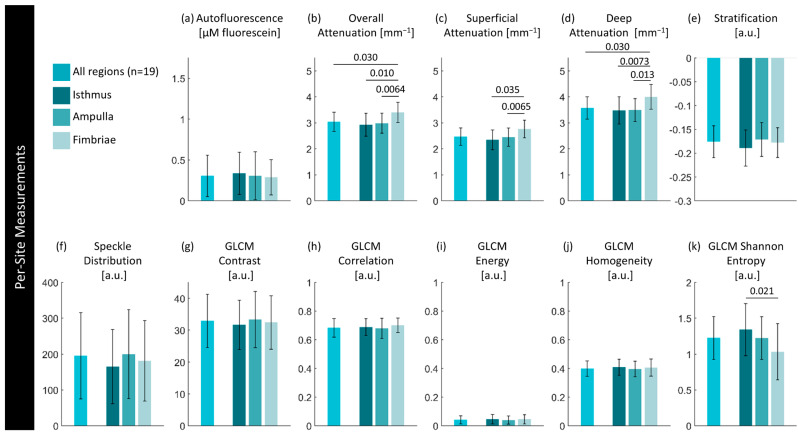
Per-site measurements of each feature per region. Values are calculated using the median measurement for each biomarker per region of interest. The height of bars represents the mean value of specimens in that group; error bars are standard deviation. *p*-Values for significant differences (*p* < 0.05) between disease states are indicated.

Visually, there is great complexity in the fallopian tubes that could result in potential confounders for the detection of cancerous changes. Figure 10 explores whether there are statistical differences in different anatomical regions (isthmus/ampulla/fimbriae) in non-lesion cases; detailed results are presented in Supplementary Table S3. In the overall superficial and deep optical attenuation measurements, there is a statistically significant increase in the median optical attenuation in the fimbriae compared to the ampulla (*p* < 0.01) or the isthmus (*p* < 0.01). There is also a significant decrease in GLCM Shannon entropy (*p* < 0.01).

In addition, we examined the correlation of these biomarkers with patient age and the time difference between arrival at the pathology department and imaging (an estimation of ischemic time). We found several slight correlations regarding age: positive correlations with the mean speckle distribution (0.604; *p* < 0.007) and autofluorescence intensity (0.670; *p* < 0.002) as well as a negative correlation with GLCM homogeneity (−0.482, *p* < 0.04). We anticipate increased fibrosis in older patients, and it follows that this would appear as an increase in autofluorescence and less homogeneity in the tissue structure. This suggests that the speckle distribution is capturing sub-resolution changes in the extracellular matrix. There were two slight correlations of attenuation coefficients with the time difference between arrival at the pathology department and imaging (overall attenuation 0.494, *p* < 0.04; superficial attenuation 0.604; *p* < 0.007). It is comforting that there are not correlations with the autofluorescence feature, indicating that our calibration approach and exclusion criteria (samples imaged >120 min after arrival) were sufficient.

Lastly, to provide a brief understanding of the variability of these biomarkers, we present a comparison of the bilateral specimens in Table 5; detailed results are presented in Supplementary Table S4. It should be noted that there may be bilateral differences in fallopian tubes, and these do not assess the reproducibility of imaging the same specimen repeatedly. There were five non-lesion specimens imaged bilaterally; one of these specimens did not include imaging of the isthmus for both the left and right fallopian tube. Table 5 presents the percentage difference between median biomarker measurements for the left and right fallopian tubes for each site. The metrics with the greatest variability between bilateral specimens containing no lesion are autofluorescence (mean percentage difference 40% overall, 51% in the fimbriae) and GLCM energy (27% overall, 28% in the fimbriae). All other measurements have a less than 10% overall difference between the left and right fallopian tubes in non-lesion specimens. 

## 4. Discussion

### 4.1. Functional Biomarkers

This work replicates our previous findings that HGSOC and STIC appear as a loss of fluorescence when examined with widefield blue excitation [56]. AFI appears similar to that of other organs with a thin layer of epithelium such as the small airways of the lung.

The overall increase in the fluorescence intensity of specimens containing a lesion was unexpected. We hypothesize that this could be capturing broader changes related to collagen remodelling or inflammation. If changes in the extracellular matrix detected with AFI are useful in tubo-ovarian cancer detection, other modalities that also examine collagen and fibrillar structures such as polarization-sensitive OCT (PS-OCT) may provide similar diagnostic utility. PS-OCT does not require double-clad fiber like OCT-AFI, and it provides higher OCT quality, which may provide better examination of structural features.

Incidentally, we found that hemosiderin deposits associated with endometriosis are strongly fluorescent. Hemosiderin has a fluorescence excitation peak at 450 nm; previous work has demonstrated hemosiderin detection using OCT and two-photon microscopy [84]. OCT-AFI may have further application beyond ovarian cancer detection, for the detection of endometriosis.

Our findings are limited by the poor signal-to-background ratio of the AFI in this system, which may result in inconsistencies in measurement regardless of calibration, as indicated by the high percentage error between the paired non-cancerous specimens. The background fluorescence contribution from the imaging system itself is comparable in scale to that of the tissue, and it varies over time—which is why we incorporate the background (air) fluorescence measurement into our calibration. Unfortunately, the background fluorescence contribution may change throughout the (sometimes minutes-long) acquisition, and taking a mean measurement at the distal exit of the specimen may not be sufficient. Reducing the background system contributions and/or introducing a control present in all cross-sections (ex. embedding fluorescent guide-stars on the catheter) may improve consistency. This approach may be preferred, as following similar calibration approaches (imaging liquid standards) may not be possible in an in vivo imaging setting as the imaging catheter will need to be sterile.

### 4.2. Attenuation Biomarkers

Previous studies have reported lower optical attenuation of non-cancerous fallopian tubes (2.5 mm^−1^) compared to our findings (3.13 mm^−1^) [41]. However, these are not measurements of the same tissue: Li et al. collected data from the exterior and serosa of the fallopian infundibula and fimbriae, whereas we collected data from within the lumen. They demonstrate similar trends of decreasing attenuation values in cancerous cases compared to non-cancerous cases; we found this to hold in our visual examination of the biomarkers, though there was minimal difference in the median value of the whole region labelled as containing lesion.

An additional concern when comparing attenuation coefficients to the literature is that they may be impacted by the confocal gate of the imaging catheter. Li et al. use a 3.8 mm diameter probe with a GRIN lens (Thorlabs GRIN2313A) with an NA of 0.23; the NA of our catheter is much lower (~0.03). Characterizing and correcting for the confocal effect of the custom imaging catheters used in this imaging study is not possible retrospectively, but in future work, this would improve the reliability and comparability of attenuation measurements.

The change in attenuation in the fimbriae was unexpected, though it does follow what Li et al. report when comparing non-cancerous infundibula to fimbriae. Given that HGSOC appears to be lower in attenuation, this should result in better contrast between lesion/non-lesion in the fimbriae where the earliest lesions originate. Depth-resolved optical attenuation may be uniquely suited as a form of tissue-specific contrast in this application.

The stratification feature seems to capture similar vessel-like structures as previously reported [40]. It also appears to capture changes in tissue layering when the endosalpinx/myosalpinx and/or myosalpinx/serosa can be visualized.

### 4.3. Texture Biomarkers

We expected the mean speckle distribution to be able to extract sub-resolution changes, and while it does appear to capture some changes such as the vessel-like structures, it is largely impeded by the birefringence artifacts and folds in tissue. There are some specimen-wide trends with respect to disease state and anatomical location, but they are hard to assess visually. While there may be future application for this feature, further work is needed to isolate diagnostically relevant contrast.

The GLCM features demonstrate reasonable potential, particularly entropy, in distinguishing HGSOC from non-lesion specimens and fimbriae from isthmus. This study only explores the barebones of what is possible here—for example, utilizing three-dimensional GLCM features may improve their ability to distinguish lesions [47].

### 4.4. Study Limitations

This work represents a small study (n = 28) with few positive cases (n = 7 cancer patients), which impacts the ability to draw firm conclusions about the biomarkers described within this paper. Instead, we present trends and potential directions for future investigation.

Recruitment and data collection took place from 2019 to 2023. Surgical delays during the COVID-19 pandemic resulted in an increase in neoadjuvant chemotherapy prior to surgery (exclusion criteria for this study) and advanced cancers that occluded the fallopian tubes, preventing cannulation. Only one out of the four HGSOC specimens in this dataset includes STIC. Imaging more specimens containing STICs is required to conclusively assess whether this approach can detect small amounts of neoplastic cells.

Data collection was conducted with a single OCT-AFI system that was not modified throughout the collection process. Retrospectively, we see several potential improvements for this device. There are methods to improve the signal-to-background ratio of the autofluorescence images (reducing plastics in the optical path, replacing components with low-fluorescence versions), which would likely improve detection of subtle features. The birefringence artifacts could be corrected through polarization diversity detection [67]. The multipath artifacts could be removed through W-type rather than double-clad fiber catheters [85], or perhaps may be leveraged to uncover additional tissue properties [86].

The segmentation approaches applied in this work are functional but could be further optimized. The deep learning tool used to identify the luminal surface would benefit from retraining on OCT-AFI from this dataset to reduce the domain transfer issues. Post-processing was required to use the predictions from this deep learning tool. The surface identification was imperfect and often included regions of mucus as tissue. We also opted to exclude regions with unreliable segmentations (e.g., due to low OCT signal in areas of birefringence artifact or overlapping reflections from the window tube and PET). Consistently including mucus and excluding regions with low signal or artifacts may have impacted our measurements. Similarly, the manual en face segmentation could be automated with its own network, which may improve consistency.

Another challenge is the precision with which diagnostic and anatomical region labels can be assessed. While we can identify lesions within the labelled regions, they are not specific, which limits our quantitative findings. Anatomical regions were estimated retrospectively and could instead have been measured during the imaging process to improve accuracy.

### 4.5. Translation and Future Directions

The eventual aim of this work is to develop a falloposcopic tool that can detect early and occult tubo-ovarian cancers in vivo prior to salpingectomy. This would be achieved through hysteroscopic cannulation of the fallopian tubes with a sterile imaging catheter under sedation. This approach has been recently demonstrated with a novel falloposcopy device of a similar size to our imaging catheter [27], though this device has additional steering and rotation capabilities which may be required for cannulation.

The imaging biomarkers measured in this work may be different in living tissue; one of the limitations of this ex vivo study is the time between resection and imaging (on average, more than an hour of ischemic time). While there were only slight correlations between imaging biomarkers (attenuation) and the time difference between arrival at the pathology department and time of imaging, we anticipate that the measurements presented in this study will not be directly translatable to in vivo tissue. In addition to ischemic time and tissue hydration impacting the ex vivo measurements, moving in vivo will introduce tissue dynamics such as blood flow and cellular metabolism.

There are several image quality improvements that could be made to the imaging device used in this study as previously described. The imaging catheters themselves are largely ready for in vivo translation, apart from a validated sterilization protocol. They are 0.9 mm in outer diameter, which fits within the working channel of a flexible hysteroscope (Olympus HYF-V). Images are acquired at a minimum pullback rate of 1 mm/s for distances up to 16 cm, allowing for imaging of the entire fallopian tube with real-time viewing of OCT and raw AFI during collection.

The imaging biomarkers described are calculated retrospectively from previously acquired OCT-AFI. Improved automation of segmentations and translation from MATLAB to a faster language (e.x. C++) could perhaps allow for viewing in the order of minutes. Additionally, not all features presented within this work may be necessary; classification approaches could be explored to identify the most diagnostically relevant features. While the features described here provide a wealth of information about the specimen, simplification may be preferable.

Novel early-detection strategies that enable fallopian tube screening may support a delay in risk-reducing definitive surgical procedures causing early menopause in high-risk patients. This requires confidence in diagnostic ability to rule out early or occult lesions, necessitating further study. Successful identification of early or occult disease via fallopian tube imaging in vivo would enable improved decision making around the timing of definitive surgery (e.g., delay for fertility preservation) and/or direct surgical procedure (e.g., more encompassing surgical staging) in patients at a general population risk or with genetic predisposition.

## 5. Conclusions

We present the first co-registered endoscopic OCT-AFI of ex vivo fallopian tubes. We present methods for the calculation of and analyze eleven imaging biomarkers for their potential to detect tubo-ovarian cancers and other lesions of interest, and conclude that there is potential in this modality, meriting further study.

Key findings include the following:
  - Autofluorescence intensity is reduced in regions of HGSOC, LGSOC, or carcinoid cancers, which can be visualized as a region of low-intensity autofluorescence co-registered with homogenous tissue in OCT.
  - The median autofluorescence is increased in specimens containing cancer compared to those with no lesions.
  - The optical attenuation coefficient is reduced in areas of lesion but increased in the fimbriae compared to the isthmus or the ampulla in non-cancerous fallopian tubes.
  - The GLCM Shannon entropy is reduced in specimens containing a cancerous lesion.
  - Hemosiderin deposits associated with endometriosis appear as intensely bright focal structures in OCT and AFI, with high optical attenuation and stratification, with reduced mean speckle distribution, and with sharp changes in GLCM features.
We also demonstrated visualization of structures in the fallopian tubes:
  - Folded and overlapping plicae resulting in subsurface gaps in OCT, including the appearance of plicae in hydrosalpinx.
  - Vessel-like structures as regions of decreased *or* increased autofluorescence compared to surrounding tissue, increased optical attenuation, stratification, and speckle distribution.
  - Regions of potential fibrotic changes as areas of high intensity OCT and autofluorescence.
  - Tissue layering suggestive of differentiable regions of endosalpinx, myosalpinx, and potentially serosa in some specimens.

## Figures and Tables

**Table 1:** Summary of biomarkers.

Category	Feature [Units]	Description	Calculation
Functional	Autofluorescence [µM fluorescein]	Intensity after calibration with respect to distance between optical core and tissue using positive (0.98 µM fluorescein) and negative (water) standards.	$0.98\;\mu M*\frac{I_{tissue}\left(d\right)-\bar{I_{background}}}{I_{brightstd}\left(d\right)-I_{darkstd}\left(d\right)}$
Attenuation	Overall Attenuation Coefficient [mm^−1^]	Mean optical attenuation coefficient over entire visualized tissue depth.	Depth-resolved method for estimating optical attenuation coefficient from OCT from Jian Liu et al. [70]: $\mu (z)\approx \frac{I(z)}{2\Delta \sum_{i=z+1}^{N}I\left(i\right)+\frac{I(N)}{\mu (N)}}$
	Superficial Attenuation Coefficient [mm^−1^]	Mean optical attenuation coefficient over upper 50% of visualized tissue depth.	
	Deep Attenuation Coefficient [mm^−1^]	Mean optical attenuation coefficient over lower 50% of visualized tissue depth.	
	Stratification [a.u.]	Ratiometric comparison of mean attenuation coefficient of superficial and deep regions. Ranges from −1 (higher deep attenuation) to +1 (higher superficial attenuation).	$\frac{\mu_{superficial}-\mu_{deep}}{\mu_{superficial}+\mu_{deep}}$
Texture	Speckle Distribution	Mean of the gamma distribution found by fitting all A-lines in the OCT cross-section.	$\frac{\alpha }{\beta }$
	GLCM Contrast	Sum of squares variance or inertia; local variations between a pixel and its adjacent neighbours in the azimuthal direction. Value of 0 represents no variation.	Haralick features calculated from the gray level co-occurrence matrix (GLCM) via MATLAB function graycoprops [46]: $\sum_{i,j}{\left|i-j\right|}^{2}p(i,j)\;$
	GLCM Correlation	Joint probability of occurrence of intensity pairs between a pixel and its neighbor. Measured from −1 (perfect negative correlation) to +1 (perfect positive correlation).	…via graycoprops [46]: $\sum_{i,j}\frac{\left(i-\bar{i}\right)(j-\bar{j})p(i,j)}{\sigma_{i}\sigma_{j}}$
	GLCM Energy	Angular second moment; uniformity of gray level distribution. Measured from 0 (no uniformity) to 1 (complete uniformity).	…via graycoprops [46]: $\sum_{i,j}{p(i,j)}^{2}\;$
	GLCM Homogeneity	Inverse difference moment; similarity between a pixel and its adjacent neighbours in the azimuthal direction. Value of 0 represents strong similarity.	…via graycoprops [46]: $\sum_{i,j}\frac{p(i,j)}{1+|i-j|}\;$
	GLCM Shannon Entropy	Randomness of the image. Value of 0 represents a completely uniform image.	MATLAB function entropy [71]: $-\sum_{i,j}p\left(i,j\right)\log_{2}p(i,j)\;$

where I is intensity; d is the distance from the optical core; μ is the optical attenuation coefficient; z is the depth in pixels in the A-Line direction; Δ is the pixel size; N is the last value of z sufficiently above the noise floor; α is the shape parameter of the gamma distribution; β is the scale parameter of the gamma distribution; p(i,j) is the value at cell (i,j) in the gray level co-occurrence matrix; σ is the standard deviation and overbars represent the mean value of a feature.

**Table 2:** Summary of statistical tests.

Statistical Question	Parametric Test	Non-Parametric Test
Is there a difference in measurements of biomarkers in volumes of different disease states?	Unpaired *t*-test [77]	Mann–Whitney U test [78]
In volumes containing a lesion, is there a difference in measurements of biomarkers within the area of lesion compared to the area of non-lesion?	Welch’s paired *t*-test [79,80]	Wilcoxon rank sum [81]
In volumes without a lesion, is there a difference in measurements of biomarkers in different regions (isthmus, ampulla, fimbriae)?	Welch’s paired *t*-test [79,80]	Wilcoxon rank sum [81]
Are there differences between the left and right fallopian tubes in patients when paired imaging is acquired?	Welch’s paired *t*-test [79,80]	Wilcoxon rank sum [81]
In volumes without a lesion, are there statistical correlations between measurements and patient age?	Spearman’s rank order [82]	
In volumes without a lesion, are there statistical correlations between measurements and time difference between arrival and imaging of the specimen?	Spearman’s rank order [82]	

**Table 3:** Dataset demographics. Values for continuous measurements are described as mean, standard error, (range).

Diagnosis		Sample Size			Age	Time to Imaging
		Left	Right	Total		
		[#]	[#]	[#]	[Years]	[Minutes]
No lesion		10	9	19	61	69
					3	5.4
					(42–81)	(23–128)
Cancerous lesions		3	4	7	65	75
					3	14.7
					(51–77)	(30–127)
	LGSOC	0	2	2	65	69
					4	6.0
					(61–69)	(63–75)
	HGSOC	3	1	4	69	80
					3	27
					(60–77)	(30–127)
	Carcinoid	0	1	1	66	66
					-	-
					-	-
Endometriosis		1	1	2	62	88
					18	2.5
					(44–80)	(85–90)
Total		14	14	28	62	72

**Table 4:** Dataset demographics for paired sets of imaging. Values for continuous measurements are described as mean, standard error, (range).

	Sample Size	Age	Time to Imaging
Diagnosis	[#]	[Years]	[Minutes]
Paired non-lesion	5	57	73
		6	7.3
		(44–80)	(49–90)

**Table 5:** Mean percentage differences in biomarker measurements between left and right fallopian tubes in paired specimens containing no lesion. Values reported are percentages.

		Functional	Attenuation				Texture					
Site	Sample Size	Autofluorescence	Overall Attenuation	Superficial Attenuation	Deep Attenuation	Stratification	Speckle Distribution	GLCM Contrast	GLCM Correlation	GLCM Energy	GLCM Homogeneity	GLCM Shannon Entropy
Overall	5	39.6	1.3	3.0	2.3	9.7	4.5	11.3	1.8	26.7	5.7	10.0
Fimbriae	5	51.4	6.4	7.7	5.8	7.3	11.8	13.8	5.2	28.8	6.2	18.9
Ampulla	5	37.3	1.7	3.5	2.6	12.7	7.8	8.4	1.2	23.1	4.7	8.4
Isthmus	4	29.8	2.1	2.9	5.3	21.0	3.4	9.1	2.2	27.9	5.6	7.4

## Data Availability

The data presented in this study are available on request from the corresponding author.

## Supplementary Materials

The following supporting information can be downloaded at https://www.mdpi.com/article/10.3390/cancers16213618/s1. Figure S1. Measurements per region of each biomarker presented in Figure 4, Figure 5, Figure 6 and Figure 7. Table S1. Inter-sample measurements of each feature per diagnosis. Table S2. Intra-sample measurements of each feature per diagnosis (lesion region vs. non-lesion region). Table S3. Measurements of each feature per site from fallopian tubes containing no lesions. Table S4. Lateral differences in biomarker measurements.
