# Supplementary material for: Multimodal Optical Imaging of Ex Vivo Fallopian Tubes to Distinguish Early and Occult Tubo-Ovarian Cancers
Source: Cancers (Basel). 2024 Oct 26;16(21):3618. doi: 10.3390/cancers16213618 (PMC11544883; doi:10.3390/cancers16213618)
Supplement: Supplementary file 1 [file cancers-16-03618-s001.zip › cancers-3226148-supplementary.pdf]

Supplementary Materials

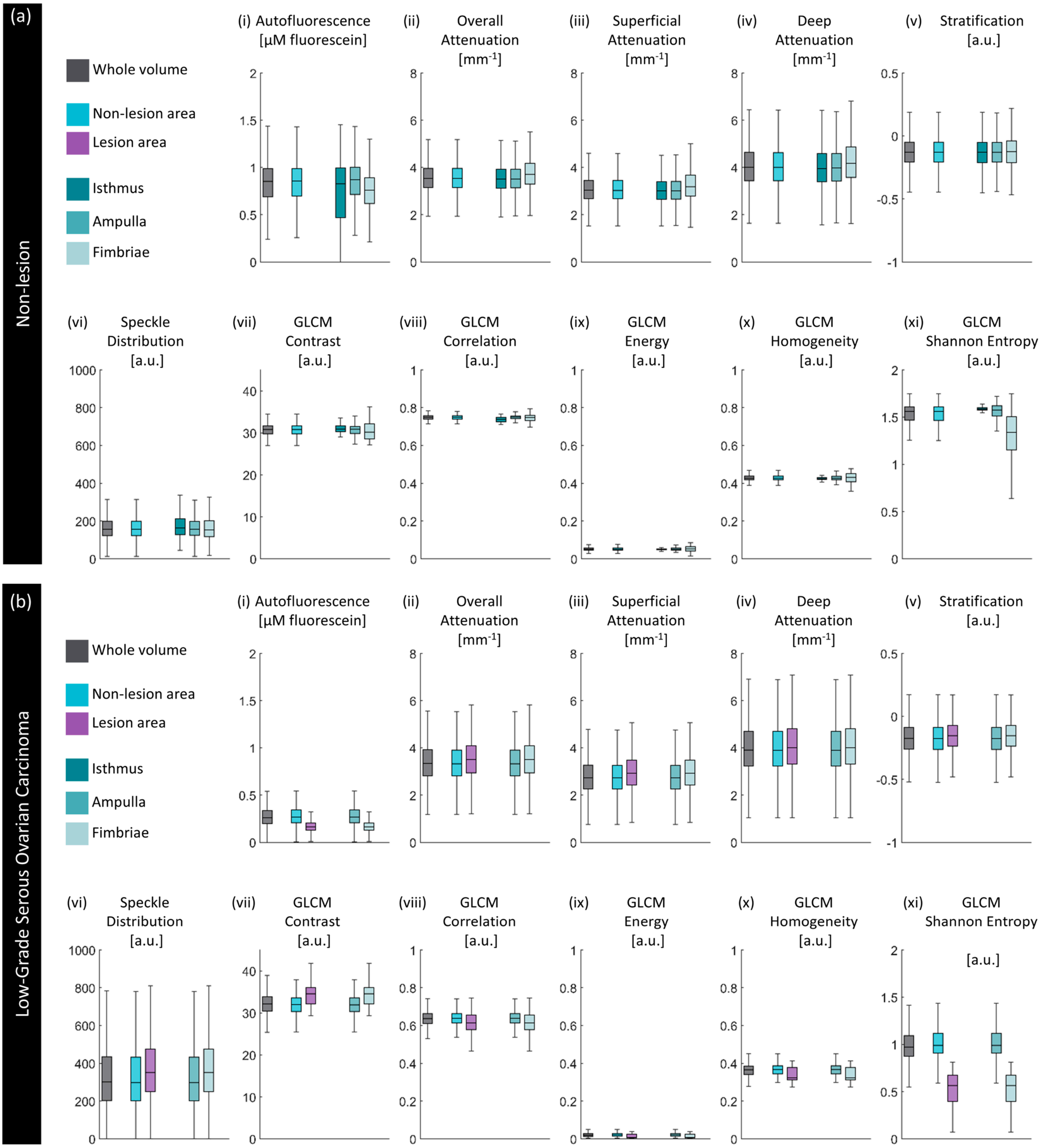

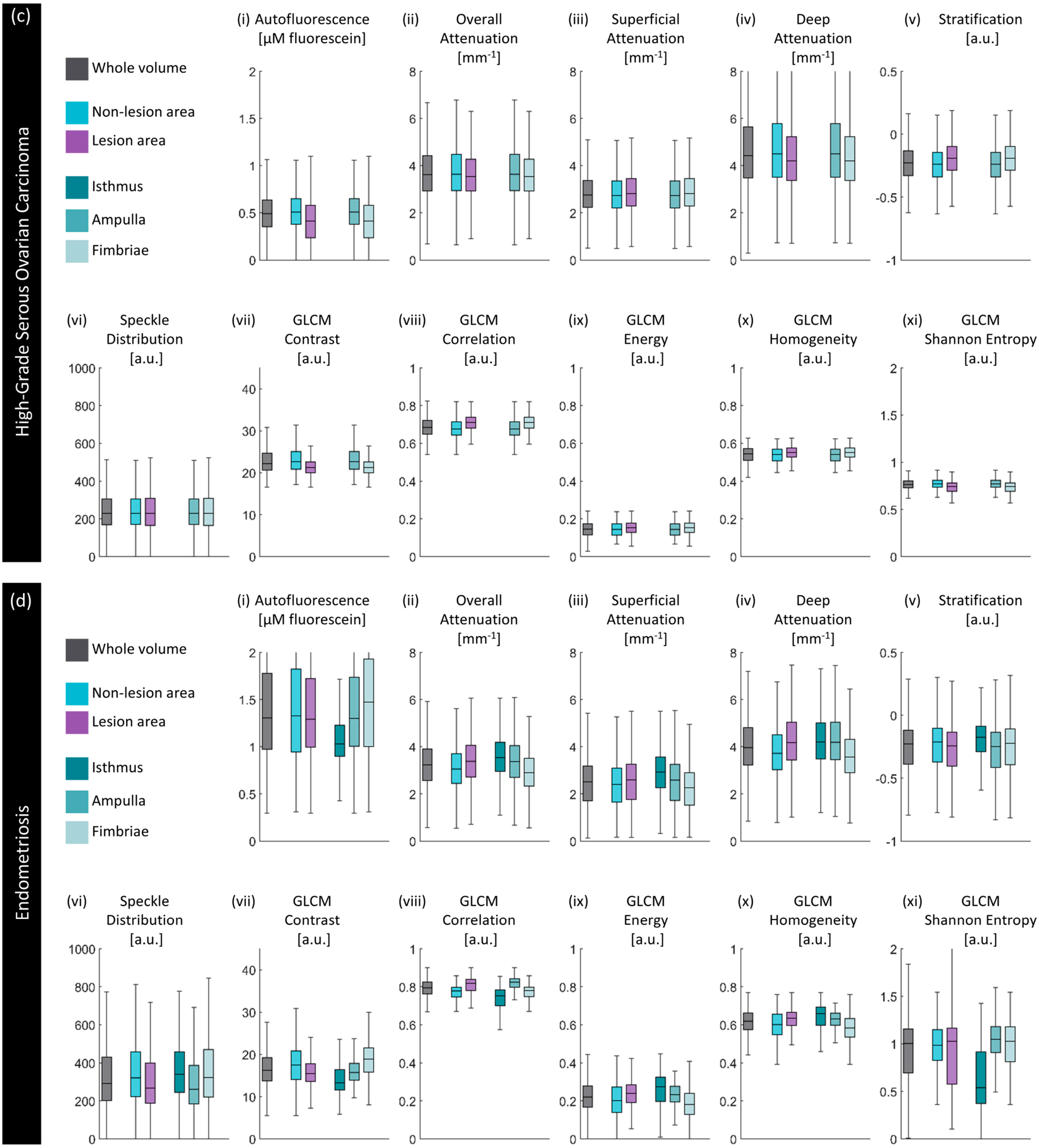

Figure S1. Measurements per region of each biomarker presented in **Figures 4–7**. Box-whisker plots demonstrate the upper and lower quartiles as the box, the median as a bar; and the maximum and minimum as whiskers. These plots compare measurements over different regions (whole volume; lesion / non-lesion; isthmus, ampulla, fimbriae).

Table S1. Inter-sample measurements of each feature per diagnosis. Data is presented as mean, standard error, (range) of the median measurement of all volumes in that category.

				Functional	Attenuation				Texture					
		**Sample size**	**Age**	**Autofluorescence**	**Overall Attenuation**	**Superficial Attenuation**	**Deep Attenuation**	**Stratification**	**Speckle Distribution**	**GLCM Contrast**	**GLCM Correlation**	**GLCM Energy**	**GLCM Homogeneity**	**GLCM Shannon Entropy**
		#	[yrs]	[µM fluorescein]	[mm^{-1}]	[mm^{-1}]	[mm^{-1}]	[a.u.]	[a.u.]	[a.u.]	[a.u.]	[a.u.]	[a.u.]	[a.u.]
Non-lesion		19	61<br>3<br>(42 – 81)	0.31<br>0.06<br>(0.10 – 1.00)	3.13<br>0.08<br>(2.29 – 3.76)	2.47<br>0.08<br>(1.73 – 3.03)	3.57<br>0.10<br>(2.66 – 4.32)	-0.18<br>0.01<br>(-0.24 – -0.12)	195<br>27.5<br>(94.5 – 445)	32.9<br>1.92<br>(20.7 – 48.0)	0.68<br>0.02<br>(0.54 – 0.77)	0.04<br>0.01<br>(0 – 0.10)	0.40<br>0.01<br>(0.31 – 0.48)	1.23<br>0.07<br>(0.61 – 1.72)
Cancers	All	7	65<br>3<br>(51 – 77)	0.58<br>0.11<br>(0.27 – 1.20)	3.36<br>0.18<br>(2.63 – 3.93)	2.68<br>0.15<br>(2.06 – 3.24)	3.82<br>0.22<br>(2.97 – 4.51)	-0.17<br>0.01<br>(-0.23 – -0.12)	181<br>24.6<br>(98.5 – 258)	26.8<br>3.69<br>(8.66 – 37.4)	0.67<br>0.01<br>(0.59 – 0.71)	0.12<br>0.06<br>(0.01 – 0.45)	0.48<br>0.06<br>(0.33 – 0.77)	0.90<br>0.11<br>(0.60 – 1.32)
	LGSOC	2	65<br>4<br>(61 – 69)	0.73<br>0.46<br>(0.27 – 1.20)	3.07<br>0.16<br>(2.91 – 3.24)	2.53<br>0.13<br>(2.39 – 2.66)	3.45<br>0.21<br>(3.24 – 3.66)	-0.15<br>0<br>(-0.15 – -0.15)	223<br>34.4<br>(189 – 258)	35.1<br>2.29<br>(32.8 – 37.4)	0.63<br>0.05<br>(0.59 – 0.68)	0.03<br>0.02<br>(0.01 – 0.04)	0.36<br>0.04<br>(0.33 – 0.40)	0.97<br>0.33<br>(0.64 – 1.30)
	HGSOC	4	69<br>3<br>(60 – 77)	0.55<br>0.04<br>(0.49 – 0.67)	3.41<br>0.30<br>(2.63 – 3.93)	2.62<br>0.21<br>(2.06 – 3.05)	3.91<br>0.36<br>(2.97 – 4.51)	-0.19<br>0.01<br>(-0.23 – -0.18)	181<br>32.6<br>(105 – 244)	27.2<br>2.81<br>(22.2 – 33.7)	0.68<br>0.01<br>(0.66 – 0.71)	0.09<br>0.03<br>(0.03 – 0.15)	0.47<br>0.04<br>(0.38 – 0.54)	0.86<br>0.16<br>(0.60 – 1.32)
	Carcinoid	1	51<br>-<br>-	0.39<br>-<br>-	3.75<br>-<br>-	3.24<br>-<br>-	4.20<br>-<br>-	-0.12<br>-<br>-	98.5<br>-<br>-	8.66<br>-<br>-	0.67<br>-<br>-	0.45<br>-<br>-	0.77<br>-<br>-	0.97<br>-<br>-
Other	Endometriosis	2	62<br>18<br>(44 – 80)	0.74<br>0.57<br>(0.17 – 1.30)	3.49<br>0.20<br>(3.29 – 3.69)	2.67<br>0.16<br>(2.51 – 2.83)	4.09<br>0.13<br>(3.96 – 4.22)	-0.21<br>0.02<br>(-0.23 – - 0.19)	178<br>85.0<br>(93.2 – 263)	19.0<br>2.75<br>(16.2 – 21.7)	0.76<br>0.04<br>(0.72 – 0.80)	0.17<br>0.05<br>(0.11 – 0.22)	0.57<br>0.05<br>(0.52 – 0.62)	1.09<br>0.09<br>(1.00 – 1.18)

Table S2. Intra-sample measurements of each feature per diagnosis (lesion region vs non-lesion region). Data is presented as mean, standard error, (range) of the median measurement of all volumes in that category.

				Functional	Attenuation				Texture					
	**Sample size**	**Age**	**Region**	**Autofluorescence**	**Overall Attenuation**	**Superficial Attenuation**	**Deep Attenuation**	**Stratification**	**Speckle Distribution**	**GLCM Contrast**	**GLCM Correlation**	**GLCM Energy**	**GLCM Homogeneity**	**GLCM Shannon Entropy**
	#	[yrs]		[μM fluorescein]	[mm^{-1}]	[mm^{-1}]	[mm^{-1}]	[a.u.]	[a.u.]	[a.u.]	[a.u.]	[a.u.]	[a.u.]	[a.u.]
LGSOC	2	65<br>4<br>(61 – 69)	Lesion	0.74<br>0.48<br>(0.27–1.22)	3.08<br>0.13<br>(2.95 – 3.20)	2.52<br>0.11<br>(2.41 – 2.64)	3.46<br>0.17<br>(3.29 – 3.63)	-0.15<br>0<br>(-0.16 – -0.15)	226<br>32.8<br>(193 – 259)	35.2<br>2.13<br>(33.0 – 37.3)	0.63<br>0.05<br>(0.58 – 0.68)	0.03<br>0.02<br>(0.01 – 0.04)	0.36<br>0.04<br>(0.32 – 0.40)	0.97<br>0.34<br>(0.64 – 1.31)
			Non-lesion	0.74<br>0.45<br>(0.30 – 1.19)	3.14<br>0.24<br>(2.90 – 3.38)	2.59<br>0.20<br>(2.38 – 2.79)	3.53<br>0.30<br>(3.22 – 3.83)	-0.15<br>0<br>(-0.16 – -0.15)	220<br>32.6<br>(187 – 252)	34.2<br>3.17<br>(31.1 – 37.4)	0.65<br>0.06<br>(0.59 – 0.70)	0.03<br>0.02<br>(0.01 – 0.04)	0.37<br>0.03<br>(0.35 – 0.40)	0.95<br>0.31<br>(0.64 – 1.27)
HGSOC	4	69<br>3<br>(60 – 77)	Lesion	0.49<br>0.04<br>(0.42 – 0.57)	3.48<br>0.29<br>(2.69 – 4.06)	2.72<br>0.23<br>(2.08 – 3.18)	3.97<br>0.33<br>(3.06 – 4.61)	-0.18<br>0<br>(-0.19 – -0.18)	174<br>31.2<br>(105 – 228)	26.7<br>2.65<br>(21.3 – 32.3)	0.69<br>0.01<br>(0.68 – 0.71)	0.09<br>0.03<br>(0.03 – 0.15)	0.47<br>0.04<br>(0.40 – 0.55)	0.80<br>0.14<br>(0.52 – 1.20)
			Non-lesion	0.59<br>0.05<br>(0.51 – 0.75)	3.35<br>0.30<br>(2.57 – 3.85)	2.57<br>0.19<br>(2.04 – 2.94)	3.87<br>0.38<br>(2.89 – 4.50)	-0.20<br>0.02<br>(-0.24 – -0.17)	185<br>32.7<br>(104 – 251)	27.4<br>2.88<br>(22.6 – 34.2)	0.68<br>0.02<br>(0.63 – 0.72)	0.09<br>0.03<br>(0.03 – 0.14)	0.47<br>0.04<br>(0.38 – 0.54)	0.89<br>0.15<br>(0.71 – 1.33)
Carcinoid	1	51<br>-<br>-	Lesion	0.34<br>-<br>-	3.88<br>-<br>-	3.34<br>-<br>-	4.31<br>-<br>-	-0.11<br>-<br>-	99.6<br>-<br>-	8.42<br>-<br>-	0.66<br>-<br>-	0.47<br>-<br>-	0.77<br>-<br>-	0.94<br>-<br>-
			Non-	0.58<br>-<br>-	3.44<br>-<br>-	2.97<br>-<br>-	3.89<br>-<br>-	-0.12<br>-<br>-	95.9<br>-<br>-	9.88<br>-<br>-	0.75<br>-<br>-	0.39<br>-<br>-	0.73<br>-<br>-	1.10<br>-<br>-

Table S3. Measurements of each feature per site from fallopian tubes containing no lesions. Data is presented as mean, standard error, (range) of the median measurement of all volumes in that category.

				Functional	Attenuation				Texture					
	Sample size	Age	Site	Autofluorescence	Overall Attenuation	Superficial Attenuation	Deep Attenuation	Stratification	Speckle Distribution	GLCM Contrast	GLCM Correlation	GLCM Energy	GLCM Homogeneity	GLCM Shannon Entropy
	#	[yrs]		[μM fluorescein]	[mm^{-1}]	[mm^{-1}]	[mm^{-1}]	[a.u.]	[a.u.]	[a.u.]	[a.u.]	[a.u.]	[a.u.]	[a.u.]
Non-lesion	19	61<br>3<br>(42 – 81)	All	0.31<br>0.06<br>(0.10 – 1.00)	3.13<br>0.08<br>(2.29 – 3.76)	2.47<br>0.08<br>(1.73 – 3.03)	3.57<br>0.10<br>(2.66 – 4.32)	-0.18<br>0.01<br>(-0.24 – -0.12)	195<br>27.5<br>(94.5 – 445)	32.9<br>1.92<br>(20.7 – 48.0)	0.68<br>0.02<br>(0.54 – 0.77)	0.04<br>0.01<br>(0 – 0.10)	0.40<br>0.01<br>(0.31 – 0.48)	1.23<br>0.07<br>(0.61 – 1.72)
			Isthmus	0.34<br>0.06<br>(0.10 – 0.92)	3.00<br>0.27<br>(2.19 – 3.82)	2.35<br>0.22<br>(1.73 – 3.01)	3.48<br>0.32<br>(2.41 – 4.33	-0.19<br>0.02<br>(-0.26 – -0.13)	165<br>26.3<br>(90.6 – 453)	31.7<br>3.17<br>(21.0 – 50.9)	0.69<br>0.06<br>(0.58 – 0.77)	0.05<br>0.01<br>(0 – 0.12)	0.41<br>0.04<br>(0.31– 0.50)	1.34<br>0.14<br>(0.61 – 1.93)
			Ampulla	0.31<br>0.07<br>(0.09 – 1.24)	3.05<br>0.09<br>(2.22 – 3.68)	2.45<br>0.08<br>(1.70 – 3.05)	3.49<br>0.10<br>(2.57 – 4.25)	-0.17<br>0.01<br>(-0.24 – -0.12)	200<br>28.4<br>(94.8 – 453)	33.3<br>2.02<br>(20.4 – 50.5)	0.68<br>0.02<br>(0.53 – 0.77)	0.04<br>0.01<br>(0 – 0.10)	0.40<br>0.01<br>(0.31 – 0.48)	1.22<br>0.07<br>(0.62 – 1.72)
			Fimbriae	0.29<br>0.05<br>(0.09 – 0.81)	3.51<br>0.09<br>(2.30 – 4.24)	2.76<br>0.08<br>(1.79 – 3.29)	4.00<br>0.11<br>(2.57 – 4.93)	-0.18<br>0.01<br>(-0.24 – -0.12)	181<br>25.7<br>(84.7 – 438)	32.4<br>1.92<br>(19.3 – 45.7)	0.70<br>0.01<br>(0.61 – 0.76)	0.04<br>0.01<br>(0 – 0.10)	0.41<br>0.01<br>(0.31 – 0.50)	1.03<br>0.09<br>(0.24 – 1.64)

Table S4. Mean percentage differences in biomarker measurements between left and right fallopian tubes in paired specimens containing no lesion. Data is presented as mean, standard error, (range) of the median measurement of all volumes in that category. Values reported are percentages.

			Functional		Attenuation					Texture			
Site	Sample size	Age	Autofluorescence	Overall Attenuation	Superficial Attenuation	Deep Attenuation	Stratification	Speckle Distribution	GLCM Contrast	GLCM Correlation	GLCM Energy	GLCM Homogeneity	GLCM Shannon Entropy
	#	[years]	[μM Fluorescein]	[mm^{-1}]	[mm^{-1}]	[mm^{-1}]	[a.u.]	[a.u.]	[a.u.]	[a.u.]	[a.u.]	[a.u.]	[a.u.]
Overall	5	57<br>6<br>(44 – 80)	39.6<br>16.6<br>(4.74 – 82.6)	1.31<br>0.980<br>(0.036 – 5.200)	2.98<br>0.962<br>(0.633 – 6.19)	2.33<br>0.716<br>(0.591 – 4.56)	9.70<br>3.21<br>(3.20 – 19.0)	4.49<br>2.01<br>(1.09 – 11.9)	11.3<br>4.82<br>(2.19 – 29.8)	1.84<br>0.651<br>(0.027 – 3.99)	26.8<br>8.30<br>(9.20 – 48.6)	5.70<br>1.31<br>(3.09 – 10.2)	9.96<br>2.60<br>(4.31 – 17.2)
Fimbriae	5	57<br>6<br>(44 – 80)	51.4<br>17.5<br>(2.01 – 83.9)	6.35<br>2.06<br>(1.36 – 11.6)	7.65<br>2.23<br>(0.956 – 13.7)	5.77<br>1.70<br>(0.804 – 9.79)	7.27<br>2.49<br>(0.746 – 14.4)	11.8<br>2.65<br>(6.08 – 20.2)	13.8<br>3.37<br>(2.04 – 22.6)	5.16<br>1.74<br>(2.05 – 11.7)	28.8<br>12.3<br>(9.47 – 77.1)	6.22<br>2.85<br>(1.50 – 17.1)	18.9<br>6.01<br>(4.40 – 37.8)
Ampulla	5	57<br>6<br>(44 – 80)	37.3<br>18.3<br>(0.669 – 83.4)	1.68<br>0.755<br>(0.374 – 4.51)	3.47<br>0.573<br>(1.81 – 5.09)	2.58<br>0.857<br>(0.614 – 4.96)	12.7<br>4.48<br>(2.33 – 24.0)	7.79<br>2.75<br>(1.69 – 14.7)	8.38<br>1.81<br>(3.80 – 14.9)	1.16<br>0.344<br>(0.424 – 2.33)	23.1<br>9.26<br>(0.725 – 49.0)	4.69<br>1.24<br>(1.92 – 7.80)	8.43<br>1.52<br>(5.55 – 13.5)
Isthmus	4	51<br>3<br>(44 – 80)	29.8<br>16.6<br>(0.974 – 64.2)	2.05<br>0.539<br>(0.513 – 2.98)	2.94<br>1.67<br>(0.027 – 5.91)	5.31<br>1.08<br>(3.17 – 8.32)	21.0<br>6.00<br>(5.85 – 33.3)	3.40<br>1.69<br>(0.033 – 8.06)	9.05<br>2.21<br>(4.84 – 15.2)	2.19<br>1.21<br>(0.073 – 5.22)	27.9<br>10.7<br>(8.94 – 53.8)	5.57<br>1.58<br>(2.02 – 8.72)	7.43<br>3.09<br>(0.565 – 15.2)
